# Electrosynthesis of high-entropy metallic glass nanoparticles for designer, multi-functional electrocatalysis

**DOI:** 10.1038/s41467-019-10303-z

**Published:** 2019-06-14

**Authors:** Matthew W. Glasscott, Andrew D. Pendergast, Sondrica Goines, Anthony R. Bishop, Andy T. Hoang, Christophe Renault, Jeffrey E. Dick

**Affiliations:** 1 Department of Chemistry, The University of North Carolina at Chapel Hill, Chapel Hill, NC 27599-3290 USA; 2 Laboratoire de Physique de la Matière Condensée, Ecole Polytechnique, CNRS, IP Paris, 91128 Palaiseau, France; 3 Lineberger Comprehensive Cancer Center, School of Medicine, The University of North Carolina at Chapel Hill, Chapel Hill, NC 27599-3290 USA

**Keywords:** Electrocatalysis, Renewable energy, Metals and alloys

## Abstract

Creative approaches to the design of catalytic nanomaterials are necessary in achieving environmentally sustainable energy sources. Integrating dissimilar metals into a single nanoparticle (NP) offers a unique avenue for customizing catalytic activity and maximizing surface area. Alloys containing five or more equimolar components with a disordered, amorphous microstructure, referred to as High-Entropy Metallic Glasses (HEMGs), provide tunable catalytic performance based on the individual properties of incorporated metals. Here, we present a generalized strategy to electrosynthesize HEMG-NPs with up to eight equimolar components by confining multiple metal salt precursors to water nanodroplets emulsified in dichloroethane. Upon collision with an electrode, alloy NPs are electrodeposited into a disordered microstructure, where dissimilar metal atoms are proximally arranged. We also demonstrate precise control over metal stoichiometry by tuning the concentration of metal salt dissolved in the nanodroplet. The application of HEMG-NPs to energy conversion is highlighted with electrocatalytic water splitting on CoFeLaNiPt HEMG-NPs.

## Abstract (web summary)

High-entropy metallic glasses are an unexplored class of nanomaterials and are difficult to prepare. Here, the authors present an electrosynthetic method to design these materials with up to eight tunable metallic components and show multifunctional electrocatalytic water splitting capabilities.

## Introduction

Complex cocktails of dissimilar metals alloyed into a single phase may present advantageous physicochemical properties, such as enhanced tensile strength^1^ and increased catalytic activity^2^. Only recently have groups started to alloy five or more elements together into a new class of materials, termed high-entropy-alloys (HEAs)^3,4^. HEAs are generally defined as containing five or more near-equimolar principle components alloyed into a crystalline solid-solution phase stabilized at an elevated mixing entropy. While the enthalpy of mixing favors phase separation to form intermetallic microstructures, materials with a homogeneous distribution of atoms can be left in a high entropy state under certain experimental conditions^5–9^. Unique properties arising from the proximal arrangement of dissimilar metal atoms, often referred to in terms of interatomic d-band ligand effects^10^ or crystal lattice strain^11^, are of great interest for generating advanced structural and functional materials. While these crystalline materials have been the subject of significant investigation^12–15^, disordered amorphous materials containing five or more components, termed High-Entropy Metallic Glasses (HEMGs), may also offer access to properties arising from dissimilar metal interactions, though these complex materials remain largely unexplored^16,17^.

Generally, high-entropy materials are formed by thermal approaches in which compositionally favorable pure metal ingots are liquefied, homogenized, and rapidly cooled to generate the bulk solid material^18^. Translating these planar bulk materials into nanoparticles (NPs) maximizes active surface–area-to-volume-ratio, which directly impacts electrocatalytic efficiency^19^. Though applying high-entropy catalytic nanomaterials to energy-relevant reactions holds great potential, the fabrication of HEA-NPs by a complex thermal-shock method was only recently reported^20^. The application of such high temperatures using these methods necessitates the use of thermally resistant substrates and may incur stoichiometric inconsistencies due to the differences in vapor pressure between molten metals. In contrast to thermal approaches, electrodeposition of metal salt precursors onto a conductive surface constitutes a room-temperature, scalable approach for the formation of amorphous metallic glass films composed of NPs^21,22^. In addition, the active surface area of electrodeposited NPs may be further enhanced by generating porous or rough particles^23^. For cathodic alloy electrodeposition experiments, variability in NP coverage, size, and stoichiometric composition stems from three main issues: preferential precursor nucleation and growth on energetically favorable sites leading to uneven surface coverage, diffusion layer overlap between neighboring NPs resulting in NP size polydispersity, and variation in precursor electrodeposition potential causing some metals to electrodeposit at higher atomic concentrations.

We recently demonstrated a solution to the issues outlined above by confining metal salt precursors to water nanodroplets suspended in dichloroethane (DCE), allowing for the isolated delivery of a specific number of precursor salt molecules to a localized nucleation and growth domain upon nanodroplet collision with a conductor^24,25^. In this method, termed nanodroplet-mediated electrodeposition, delivery of the precursor atoms to the substrate results from the formation of a ~10 nm droplet/electrode contact radius upon nanodroplet collision, which we recently exploited to observe the nucleation and growth of single Pt NPs in real time on ultramicroelectrodes (UMEs)^26^. In these experiments, we determined that elevated mass transfer (i.e., rapid reduction of chloroplatinate to Pt^0^) within sub-femtoliter (10^−15^ L) nanodroplets played an integral role in the formation of porous Pt NPs. Interestingly, a 55-ms carbothermal-shock followed by rapid quenching (10^5^ K/s) played an integral role in the first reported fabrication of HEA-NPs by Hu et al. in 2018^20^.

Extending this concept, here we use nanodroplet-mediated electrodeposition to demonstrate that the collision of nanodroplets with a biased electrode represents an electro-shock event on the order of 100 ms, rapidly reducing up to eight confined metal salt precursors into HEMG-NPs with precisely tunable stoichiometric ratios. Furthermore, we use this method to design a CoFeLaNiPt HEMG-NP electrocatalyst for complete water electrolysis to demonstrate the multi-functional potential of this synthetic method.

## Results

### Electrosynthesis and characterization of HEMG-NPs

**Fig. 1:**
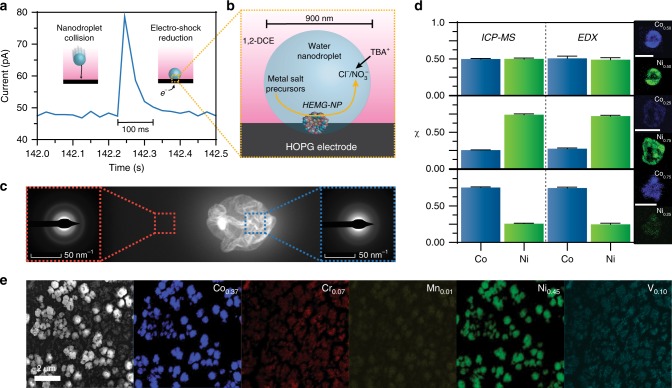
Nanodroplet-mediated electrodeposition overview for controlling NP stoichiometry and microstructure. **a** Current transient corresponding to the collision of a single nanodroplet onto a carbon fiber UME (*r*_UME_ = 4 μm) biased at −0.4 V vs. Ag/AgCl. Nanodroplet contents are fully (>98%) reduced within 100 ms, facilitating disordered co-deposition of various metal precursors. **b** Representation of a nanodroplet collision event highlighting the rapid NP formation at the water/substrate interface and the charge balance ensured by the transfer of TBA^+^ across the oil/water interface. **c** An amorphous microstructure is confirmed by a lack of crystallinity at high resolution and the presence of diffuse rings on the SAED pattern. **d** Correlated ICP-MS and EDX results on Co0.5Ni0.5, Co0.25Ni0.75, and Co0.75Ni0.25 MG-NPs confirming precise control over NP stoichiometry. **e** Alloy film electrodeposited from aqueous solution of equimolar metal salt precursors demonstrating phase and stoichiometric heterogeneity. Source data for panels **a** and **d** are provided in the Source Data file

Figure 1a shows a representative collision of a single nanodroplet filled with 8 mM CoCl_2_, MnCl_2_, CrCl_3_, NiCl_2_, and VCl_3_ (40 mM of metal salt in total) onto a carbon fiber UME (*r*_UME_ = 4 μm). The minimal background current provided by the UME due to its micrometer dimensions facilitates the observation of single nanodroplet collision events. After an initial sharp rise corresponding to the collision of the nanodroplet with the UME, the current decays due to the consumption (electrolysis) of the metal salt initially contained inside the nanodroplet^27,28^. Importantly, the width of the cathodic peak indicates that electrodeposition is finished within *ca*. 100 ms, a timescale similar to the one encountered in the carbothermal-shock synthesis (55 ms). The amount of charge transferred during the reduction process, *Q*_red_, is obtained by integrating under the blip-type response shown in Fig. 1a. The analysis of multiple independent cathodic peaks leads to an average value of *Q*_red_ = 3.47 ± 2.68 pC. This amount can be compared to the initial amount of metal precursor contained within a nanodroplet and the corresponding charge, *Q*_ini,_ required to reduce all the precursor ions. According to Faraday’s Law, this charge is given by Eq. 1.

1 $$Q_{{\mathrm{ini}}} = \frac{4}{3}\pi Fr_{\mathrm{drop}}^3\mathop {\sum }\nolimits^k _{i=1} n_iC_i$$

where *F* is Faraday’s constant, *r*_drop_ is the average radius of a nanodroplet, *n*_i_ is the number of electrons involved in electron transfer for the salt *i*, and *C*_i_ is the concentration of the salt *i*. Using an average value of *r*_drop_ = 450 nm, obtained by previous dynamic light scattering and nanoparticle tracking analysis results^26^, we calculated a value of *Q*_ini_ = 3.53 pC (*Q*_red_/*Q*_ini_ = 98.2%). We conclude that all the metal ions initially contained in the nanodroplet are effectively reduced upon collision with an electrode biased at −0.4 V vs. Ag/AgCl (Fig. 1b). For the remainder of this work, we use a higher cathodic potential (−1.5 V vs. Ag/AgCl) to ensure that electrodeposition occurs at or near the mass transfer limit for each depositing species.

Transmission electron microscopy (TEM) was used to investigate the microstructure of binary CoNi NPs electrodeposited at equal stoichiometric ratios (Fig. 1c), giving a selected area electron diffraction (SAED) pattern with diffuse rings characteristic of amorphous materials^29,30^. Probing the atomic distribution within the amorphous microstructure by energy dispersive X-ray spectroscopy (EDX) revealed a disordered cobalt-nickel arrangement. In this work, we demonstrate an important feature of the electro-shock synthesis: precise tuning of NP stoichiometry can be achieved by modulating the ratio of the confined metal salt within water nanodroplets. By adding 20 mM CoCl_2_ and 20 mM NiCl_2_ to the nanodroplets and analyzing the composition of the resulting NPs electrodeposited onto highly oriented pyrolytic graphite (HOPG) at −1.5 V vs. Ag/AgCl with EDX and inductively coupled plasma mass spectrometry (ICP-MS), we confirmed the stoichiometry of Co_0.5_Ni_0.5_ was in agreement with the initial stoichiometry of the metal salt dissolved in the nanodroplet. Tuning the proportion of CoCl_2_ and NiCl_2_ facilitated the generation of Co_0.25_Ni_0.75_ and Co_0.75_Ni_0.25_ MG-NPs with high precision, which were also verified by ICP-MS (Fig. 1d, Supplementary Table 1). Importantly, ICP-MS data were perfectly aligned with EDX quantification despite the greater uncertainty in EDX measurements^31^, as indicated in Fig. 1d. These results stand in stark contrast to the stoichiometric variation within a CoCrMnNiV film electrodeposited from an aqueous solution containing 20 mM aliquots of each precursor metal salt onto a glassy carbon electrode biased at −1.5 V vs. Ag/AgCl. The resulting EDX maps reveal a preferential co-deposition of a CoNi-rich phase and separate CrMnV phases (Fig. 1e). Using nanodroplet-mediated electrodeposition, the stoichiometry of the CoCrMnNiV NPs can be controlled to near-equimolar ratios, as shown by EDX and validated by ICP-MS, maximizing the mixing entropy (Supplementary Fig. 1). Typical electrodeposition and electroplating methods employ chemical additives, such as polyethylene glycol or hydrochloric acid, to obtain a desirable microstructure, which must be optimized based on the metal salt composition^32^. In this generalized method, confining metal salts to water nanodroplets appears to mitigate the phase-separation effects, as NPs formed by electro-shock reduction maintain the disordered structure and intended stoichiometry shown by EDX and ICP-MS over a wide variety of metal compositions (vide infra). It is worth noting that typical lithographic methods used to generate complex alloy nanomaterials incur up to 50% variability in stochiometric control^33^, whereas this method allows precise stochiometric control within 2–5%. Furthermore, the ICP-MS results validate EDX as a robust semi-quantitative method for determining approximate NP stoichiometries. These results demonstrate the successful electrosynthesis of disordered MG-NPs and are in agreement with the literature for the formation of mixed-metal oxide films of CoNi^34,35^. Alloys in this work are discussed in terms of the base metal without regard for the oxidation state (i.e. CoNi). X-ray photoelectron spectroscopy (XPS) data found in Supplementary Figs. 2–3 detail oxidation states of metals in CoFeLaMnNi and CoFeLaNiPt HEMG-NP systems, revealing that all metal species exist as oxides under the conditions necessary for XPS analysis. While these data are useful in indicating the presence and ambient oxidative condition of the HEMG material, they do not necessarily represent the oxidation state of these materials under an applied bias in solution. We are currently investigating the in situ oxidation states of NPs using surface-interrogation scanning electrochemical microscopy^36,37^.

**Fig. 2:**
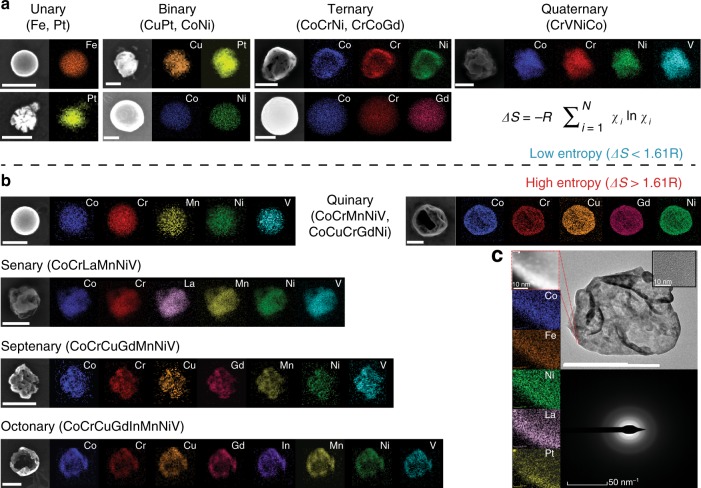
Elemental and microstructural characterization of alloy NPs with up to eight components. **a** EDX maps of various low-entropy MG-NPs. A low-entropy classification is dictated by <4 elemental components with equimolar stoichiometric ratios to give a ΔS < 1.61 R. **b** EDX images of HEMG-NPs (ΔS > 1.61 R) produced by adding up to eight metal salt precursors to the nanodroplets. **c** HAADF-STEM images of a CoFeNiLaPt HEMG-NP with accompanying high-resolution EDX images showing disordered elemental distribution at the atomic scale. The diffuse rings on the SAED pattern indicate an amorphous microstructure. All NPs were electrodeposited at −1.5 V vs. Ag/AgCl. Scalebars represent 500 nm unless otherwise indicated

The scope of the nanodroplet electro-shock method was demonstrated by loading nanodroplets with various combinations of metal salt precursors and showing their atomic arrangement and semi-quantitative stochiometric ratio in the resulting NP by EDX. Figure 2a shows the resulting SEM and EDX images obtained over MG-NPs with up to four elemental components, constituting low-entropy systems with ΔS < 1.61 R^38^. Figure 2b shows SEM and EDX images obtained for various HEMG-NP systems, which have a calculated mixing entropy ΔS > 1.61 R. High-angle annular dark-field scanning transmission electron microscopy (HAADF-STEM) and SAED were used to validate the conservation of a disordered, amorphous microstructure in this high-entropy system (Fig. 2c). The high resolution of HAADF-STEM EDX mapping (<20 nm) showing a disordered atomic arrangement supports the lower resolution EDX images obtained by SEM. In addition, X-ray diffraction (XRD) was employed to support the amorphous microstructure from SAED (Supplementary Fig. 4). Semi-quantitative atomic percentages via EDX obtained over three particles for each alloy configuration are presented in Supplementary Table 2, confirming maximized entropy and demonstrating stoichiometric control based on the concentration of metal salt precursor added to the nanodroplets.

Some of the metals incorporated into these NPs, such as manganese, have been observed to electrodeposit poorly under general aqueous conditions^39^. Characterization of individual metal salt species electrodeposited on HOPG during cyclic voltammetry (CV) are shown in Supplementary Fig. 5. While these data are useful for determining an approximate onset potential for nucleation and growth onto the carbon substrate, this evaluation may not accurately represent the kinetics of alloy formation due to the previously reported favorable thermodynamic shift associated with co-deposition of multiple metals^40^. Furthermore, our previous results (Fig. 1) comparing nanodroplet-mediated electrodeposition to bulk aqueous electrodeposition, indicate stoichiometric control over systems where precise control has been traditionally difficult to achieve.

### Design and application of a multifunctional HEMG-NP electrocatalyst

We demonstrate the versatility of the electro-shock method and its application to designer, multifunctional electrocatalysis by synthesizing a novel CoFeLaNiPt HEMG-NP electrocatalyst for cathodic and anodic water splitting. Generally, electrocatalysts are synthesized and optimized for a particular electrocatalytic reaction. However, it may be economically beneficial to design nanomaterial electrocatalysts amenable to multiple electrocatalytic reactions, a task which is streamlined by the synthetic method presented in this work. For instance, two important reactions for the production of fuel cell reactants are the oxygen evolution reaction (OER) and the hydrogen evolution reaction (HER). Although Pt^41^ offers facile kinetics for the HER, it displays rather poor performance for the OER. Furthermore, metal oxides (MO_x_) and oxyhydroxides, such as CoO_x_^42^, FeO_x_^43^, NiO_x_^44^, and mixed MO_x_, such as NiFeO_x_^45^, have been shown to be kinetically favorable toward the OER; however, each of these MO_x_ compounds have poor activity toward the HER. Rare-earth oxides, such as La_2_O_3_, have been used to confer stability to materials exposed to alkaline conditions^46^. The literature precedent surrounding these materials inspired us to design a CoFeLaNiPt HEMG-NP electrocatalyst amenable to the OER and the HER at a single nanomaterial. It is important to note that the calculated entropic contribution to the total Gibb’s free energy of the NP arises from near-equimolar elemental concentrations, whereas colloquial alloy systems generally contain a single principle element doped with other metals in low concentration^47^. Electrocatalytic activity may be greatly amplified by tuning the stoichiometric ratios, as was reported for the record oxygen evolution activity of Fe_0.25_Ni_0.75_ oxyhydroxide materials^48^. Because the high-entropy classification is predicated on the equimolar ratio of the elemental constituents, it may be necessary to tune metal ratios away from the high-entropy state to optimize electrocatalysis. In this work, it is our goal to explore the near-equimolar synthesis of HEMG-NPs using nanodroplet-mediated electrodeposition and demonstrate the precise tuning of metals rather than to search for an optimal stoichiometric ratio for electrocatalytic water splitting.

**Fig. 3:**
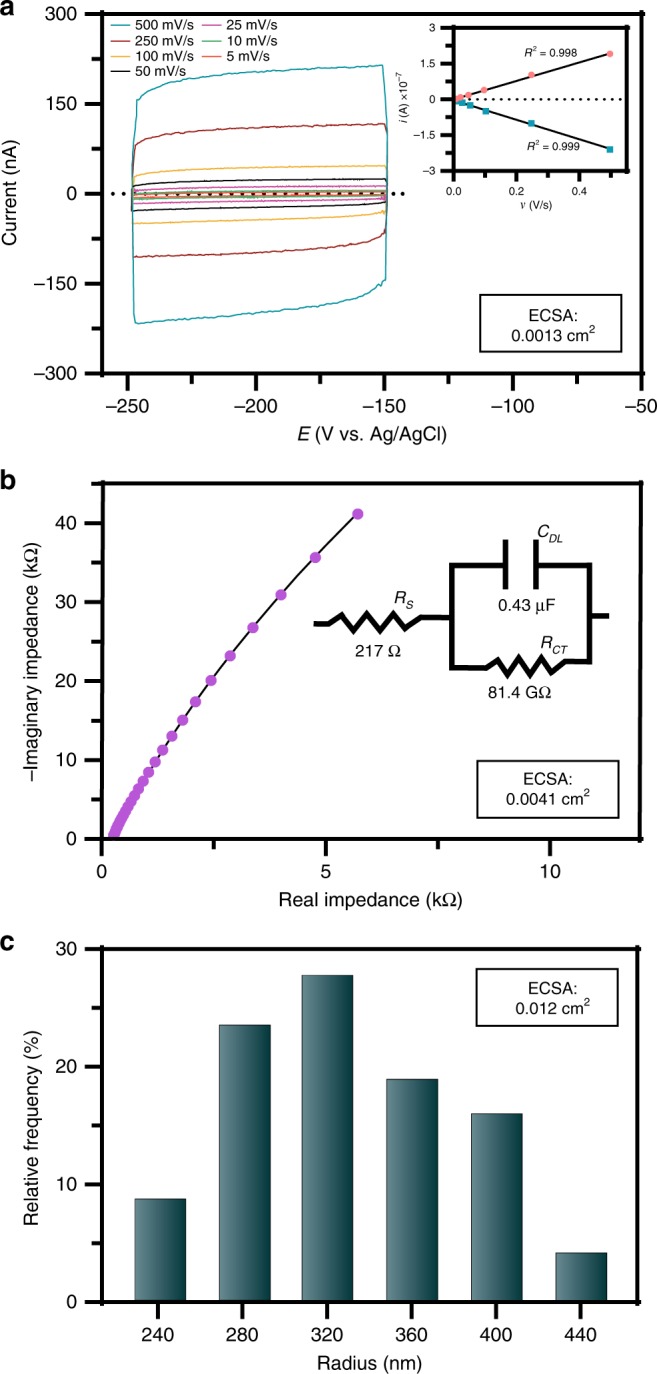
Evaluation of catalyst electrochemically active surface area. **a** CV analysis on the CoFeLaNiPt HEMG-NP electrocatalyst from −0.05 V to 0.05 V vs. OCP (−0.25 to −0.15 V vs. Ag/AgCl) at 5, 10, 25, 50, 100, 250, and 500 mV/s in 0.1 M KOH. The inset plot shows the cathodic and anodic current contributions as a function of scan rate. **b** Nyquist plot obtained by EIS fitted to the Randles cell (inset). **c** Histogram of observed HEMG-NP radius in SEM. The background capacitance of the bare HOPG electrode was subtracted from the obtained double layer capacitance to compensate for the low substrate coverage

Accurate determination of the electrochemically active surface area (ECSA) for a given material is extremely important to quantify and compare electrocatalytic activity. Commonly, the double layer capacitance of the catalyst is determined by CV in a non-faradaic region or by electrochemical impedance spectroscopy (EIS)^49^. After electrodeposition of CoFeLaNiPt HEMG-NPs onto the HOPG substrate at −1.5 V vs. Ag/AgCl, CV at seven scan rates was conducted (Fig. 3a). Plotting the cathodic and anodic current as a function of the scan rate revealed a linear function, where the slope indicated the double layer capacitance (C_DL_). EIS can also be used to obtain the double layer capacitance by fitting the impedance response of the system at different frequencies to the Randles circuit (Fig. 3b). The double layer capacitance can be correlated to the ECSA using the specific capacitance of the material, and further details of this analysis can be found in the Materials and Methods. Using CV and EIS, we determined the ECSA of the CoFeLaNiPt HEMG-NPs to be 0.0013 and 0.0041 cm^2^, respectively. Nanodroplet-mediated electrodeposition offers an additional metric for approximating the ECSA through an integrated form of the Cottrell equation to determine the total number of NPs incident on the electrode surface over a certain deposition time (*t*), given by:

2 $${\mathrm{Coverage}}\left( {\frac{{NPs}}{{m^2}}} \right) = 2C^ \ast D^{\frac{1}{2}}\pi ^{ - \frac{1}{2}}t^{\frac{1}{2}}N_A$$

where ${C^{\ast}}$ is the concentration of nanodroplets suspended in DCE, *D* is the nanodroplet diffusion coefficient as determined by the Stokes-Einstein relationship, and *N*_*A*_ is Avogadro’s number. This analysis approximates NP coverage based on diffusional mass transfer of nanodroplets to the electrode surface with subsequent electrodeposition to form a single NP, which was previously validated for Pt NPs electrodeposited from water nanodroplets^23^. By assuming a hemispherical geometry and using the average NP radius of 320 nm determined by SEM, an approximate ECSA was calculated as 0.012 cm^2^, as shown in Fig. 3c. For the analysis of our electrocatalytic materials, we used the average of the ECSA values determined by these three methods (Supplementary Figs. 6–17).

**Fig. 4:**
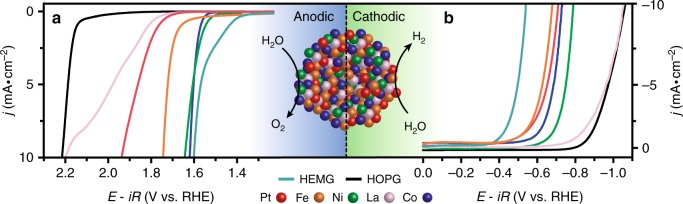
Electrocatalytic evaluation of a CoFeLaNiPt HEMG-NP electrocatalyst. **a** Anodic polarization of the HEMG electrocatalyst and its individual components starting from the equilibrium potential of OER, 1.23 V vs. RHE. Each material was loaded onto the HOPG substrate by nanodroplet-mediated electrodeposition. The current was converted in terms of the current density, *j*, based on the ECSA. **b** Cathodic polarization of the HEMG electrocatalyst and its individual components to drive HER starting from 0 V vs. RHE. All measurements were temperature controlled to 25 °C, purged with O_2_ for OER and H_2_ for HER, and *iR* corrected. For convenience, we represented the OER and HER on the same NP, but these two reactions are not carried out simultaneously. Source data for panels **a** and **b** are provided in the Source Data file

We chose to evaluate these electrocatalysts in 0.1 M KOH due to increased metal oxide stability^50^, reduced cationic interaction^51^, and practicality for real-world water electrolysis applications^52^. Resulting *iR*-corrected linear sweep voltammograms at 10 mV/s given in Fig. 4 highlight HEMG-NP functionality for both the HER and the OER. Electrocatalyst overpotential (*ƞ*) is most often reported as the difference between the applied potential (*E*) and the equilibrium potential (*E*_eq_), which is 1.23 V vs. RHE for the OER and 0 V vs. RHE for the HER^53^. Using this metric at a current density of 10 mA cm^−2^, we obtained an OER overpotential of 377 ± 4 mV for the HEMG-NPs. Significantly, this finding represents a 139 and 36 mV improvement over unary Fe and Ni, respectively, demonstrating synergistic transition metal oxide interactions^54^. The HEMG-NP overpotential also corresponds to typically reported values for IrO_x_, a benchmark OER electrocatalyst^55^. For the HER, the HEMG-NPs significantly outperformed the individual components with an overpotential of 555 ± 2 mV, representing an improvement of 132, 176, and 183 mV over Fe, Pt, and Co NPs, respectively. Though these HER overpotentials are slightly elevated compared to the current literature, the relative activity of each metal component is conserved^53^. Assuming platinum as the active site, this observation may be explained by elemental synergisms between Pt and one or more of the other elemental components on the atomic scale, or possibly an increase in Pt surface area based on its atomic distribution in the amorphous microstructure. Additionally, a bulk polycrystalline Pt disk electrode was evaluated in 0.1 M KOH to verify the efficacy of the system for the HER, and the obtained overpotential of 201 ± 15 mV agrees with literature values (Supplementary Fig. 18)^53^. The overpotential necessary to drive the HER on these electrodeposited nanomaterials is significant and possibly indicates active site poisoning. Therefore, while this work demonstrates the potential for the electro-shock method to generate novel electrocatalysts, increasing the overall electrocatalytic efficiency of these materials requires optimization. The HEMG-NP electrocatalyst was found to be stable for at least 1 h at an overpotential necessary to produce 10 mA cm^−2^ for both the OER and the HER (Supplementary Fig. 19). Furthermore, metal loading on the substrate as a result of the nanodroplet-mediated electrodeposition method was on the order of 10 ng cm^−2^ for each material, constituting a significant reduction in mass compared to typically employed water splitting electrocatalysts. Other electrocatalytic parameters derived from this analysis, such as mass activity and Tafel slope, can be found in Supplementary Table 3. In this work, it is our intent to showcase a generalized platform for the design and synthesis of novel catalytic multi-component materials. To this end, we present the CoFeLaNiPt material as a successful example of an electrocatalyst specifically designed for both the HER at Pt active sites and the OER at transition metal active sites, which happens to show interesting synergistic activity above that of its individual components. In principle, the ability to functionally and stoichiometrically tune activity by varying metal salt species and ratios within water nanodroplets offers exciting opportunities for many electrocatalytic systems.

## Discussion

HEMG nanomaterials present an opportunity to enhance a catalyst’s chemical and physical properties by selectively incorporating particular elements in certain ratios. In this report, we have presented a facile one-step method, nanodroplet-mediated electrodeposition, to fabricate HEMG-NPs with up to eight principle metallic components while maintaining precise control over elemental stoichiometry. Analysis of single emulsion nanodroplet collision events at a carbon fiber UME indicated complete electrolysis on the order of 100 ms, facilitating rapid NP formation. This electro-shock reduction generated near-equimolar NPs characterized by a disordered, amorphous microstructure confirmed by HAADF-STEM, SAED, and XRD. High-resolution EDX analysis of single NPs demonstrated homogeneous atomic distribution, a property that may elucidate functional synergism towards electrocatalysis. The CoFeLaNiPt HEMG-NP system offers multi-functional activity for the HER and the OER by combining desirable electrocatalytic properties of transition and noble metals, and this generalized synthetic platform represents a unique opportunity to tune this class of HEMG-NP electrocatalysts for a wide range of reactions.

## Methods

### Reagents and materials

All chemicals were of analytical grade unless noted otherwise and used as received. Dichloroethane (DCE, 99.8%), tetrabutylammonium perchlorate (TBAP, 99%), sodium dodecyl sulfate (SDS, 99%), hexachloroplatinic acid, and iron (III) chloride were obtained from Sigma-Aldrich and used without further purification. Chromium (III) chloride, cobalt (II) chloride, manganese (II) chloride, vanadium (III) chloride, nickel (II) chloride, indium (III) chloride, cobalt (II) nitrate, nickel (II) nitrate, lanthanum (III) nitrate, iron (II) nitrate, platinum (IV) nitrate, and gadolinium (III) chloride were obtained from Fisher Scientific and used without further purification. Stock solutions (100–300 mM) of each metal were prepared in MilliQ water (>18 MΩ cm) and diluted as necessary to be used in the emulsion preparation. Metal salt solutions were stored in a dark refrigerator (4 ℃) to avoid photodecomposition. Metal salt compatibility was investigated to prevent competing reactions (Supplementary Table 4). Metal salts were analyzed by UV-vis spectroscopy to ensure no leakage into the DCE phase following sonication (Supplementary Fig. 20).

### Instrumentation

The electrodeposition experiments were performed using a CHI model 601E potentiostat (CH Instruments, Austin, TX) using the Amperometric *i*–*t* Curve technique. Rotating disk electrolysis and electrocatalytic analysis were performed with a WaveDriver 200 and WaveVortex (Pine Instruments, Durham, NC). A 3-mm radius glassy carbon rod was used as the counter electrode. To decrease the risk of silver leakage from the reference electrode, we employed a double-junction Ag/AgCl electrode modified with a home-built 3.5 M KCl, 5% *w/w* agarose salt bridge. No obvious junction potential or other deleterious effects could be observed in the cyclic voltammogram of 1 mM ferrocene methanol in a 0.1-M KCl aqueous solution as a result of the double-junction reference. In addition, we have found this material to be stable in DCE for over two months. The emulsion was prepared using a Q500 ultrasonic processor (Qsonica, Newtown, CT) with a ¼” microtip probe. A V-650 UV-vis Spectrometer (Jasco, Oklahoma City, OK) was used to quantify metal salt leakage from the aqueous emulsion phase to the continuous DCE phase. SEM images and EDX spectra were acquired using a Helios 600 Nanolab Dual Beam System (FEI, Hillsboro, OR) and INCA PentaFET -x3 (Oxford, Abingdon, United Kingdom), respectively, at 30 keV and 0.69 nA. TEM images and diffraction patterns were collected using a 2010F-FasTEM (JEOL, Peabody, MA) with a zirconated tungsten thermal field emission tip at 200 keV. X-ray diffraction (XRD) spectra were collected using a Rigaku SmartLab X-ray diffractometer (Rigaku, Tokyo, Japan) in a 2*θ* grazing angle configuration. Inductively coupled plasma mass spectrometry (ICP-MS) data were collected using a Nexio 300-D ICP-MS (Perkin Elmer, Waltham, MA). HAADF-STEM and diffraction images were collected with a Talos F200X G2 (FEI, Hillsboro, OR) at 200 keV using a single-tilt holder.

### Nanodroplet-mediated electrodeposition procedure

HEMG-NPs were electrodeposited on HOPG or glassy carbon substrate electrodes from a water-in-oil emulsion system, as previously described^56^. In brief, a 30-µL aqueous phase containing a total of 40 mM metal salt precursor ions was suspended in a 5-mL DCE continuous phase. The application of a chloroform or ionic liquid continuous phase has also been explored^57,58^. TBAP (0.1 M) was added to the DCE phase as a non-aqueous supporting electrolyte and charge balance mediator. The resultant two-phase solution was subsequently ultrasonicated (500 W, amplitude 40%) using a pulse mode method (5 s on, 5 s off, 6 total cycles) for emulsion formation. Following sonication, a three electrode electrochemical cell was inserted into the emulsion and biased at a potential sufficient to reduce metal salt precursors at the electrode surface (−1.5 or −0.4 V vs. Ag/AgCl, 3 mm glassy carbon counter electrode). Individual current transients for attoliter nanodroplet collisions were recorded following diffusion and subsequent reduction at a carbon fiber UME. For NP morphology and elemental composition characterization experiments, the electrodeposition was carried out on a 3-mm HOPG disc electrode secured in a Teflon cell with a Viton O-ring. The substrate was exfoliated and rinsed with ethanol prior to emulsion loading, and the post-deposition substrate was rinsed with ethanol and water prior to characterization.

### Electron microscopy and energy dispersive spectroscopy analysis

Single HEMG-NP images and energy dispersive spectroscopy (EDX) spectra were acquired using a Helios 600 Nanolab Dual Beam System (FEI, Hillsboro, OR) and INCA PentaFET -x3 (Oxford, Abingdon, United Kingdom), respectively, at 30 keV and 0.69 nA. High resolution elemental mapping for semi-quantitative stoichiometric analysis of individual HEMG-NPs was collected at 30 keV and 11 nA for up to 10 min of continuous EDX scanning to enhance signal-to-noise ratio while minimizing drift in the SEM. EDX maps were brightness and contrast adjusted and false-colored to demonstrate homogeneous elemental distribution within single HEMG-NPs. Atomic resolution HAADF-STEM/EDX maps were generated using a SuperX Energy Dispersive Spectrometry system (SuperX EDX) with four Silicon Drift Detectors (SDD). Molybdenum grids coated with amorphous carbon (Ted Pella, Redding, CA) were used to support HEMG-NPs for TEM analysis to avoid competing Cu signal during EDX analysis.

### X-ray photoelectron spectroscopy and X-ray diffraction analysis

XPS spectra were obtained under UHV with a base pressure of 5 × 10^−9^ torr on a Kratos Axis Ultra DLD with a monochromatic Al kα source coupled with a hemispherical analyzer. High resolution XPS spectra were baseline subtracted with a Shirley background correction method for subsequent individual peak fitting and peak identification. High coverage HEMG-NP samples were electrodeposited on a glassy carbon rotating disk electrode (*r* = 2.5 mm) at 1000 rpm and −1.5 V vs. Ag/AgCl and subsequently rinsed with ethanol and water prior to analysis. High substrate coverage was confirmed via SEM prior to surface characterization by XPS. XPS survey scans indicated the presence of characteristic carbon and oxygen peaks consistent with the underlying glassy carbon substrate electrode. XPS survey scans and high-resolution element scans are presented in the supplementary information for two representative quinary HEMG-NP systems indicating the presence of metal oxide species. XPS peak identification of high resolution regions was achieved by comparing binding energy and peak splitting values with standard literature values. These high coverage samples were subsequently analyzed with a Rigaku Smartlab XRD in a 2*θ* grazing angle orientation to confirm the amorphous microstructure.

### Inductively coupled plasma mass spectrometry analysis

Stoichiometric analyses for high coverage HEMG-NP samples were obtained using a Nexio 300-D ICP-MS to supplement EDX analysis for single NPs. High coverage CoNi HEMG-NP samples were prepared by electrodeposition on a glassy carbon rotating disk electrode (*d* = 5 mm) at 1000 rpm and −1.5 V vs. Ag/AgCl. NPs were extracted with concentrated nitric acid and diluted to 5% *v/v* acid matrix in water prior to ICP-MS analysis.

### Electrocatalytic analysis

Electrocatalytic analysis was carried out using a Pine WaveDriver 200 and AfterMath software in a temperature-controlled (25 °C) Teflon housing actively purged with H_2_ (HER) or O_2_ (OER). The pH of 0.1 M KOH was confirmed before each characterization at 13.00 ± 0.01. After material deposition, EIS at an AC amplitude of 20 mV was conducted about the OCP from 100 kHz to 10 Hz in order to determine the double layer capacitance. The uncompensated resistance of the 0.1 M KOH electrolyte solution was obtained using a pre-programed EIS method and was found to be 217 ± 29 Ω over 13 experiments (Supplementary Fig. 21). In addition, because the absolute current passed during electrocatalytic evaluation was generally around 50 μA, the margin of error introduced by this uncompensated resistance amounts to less than 10 mV. The uncompensated resistance was accounted for at a correction factor of 85% for all voltammetric measurements. CVs in the non-faradaic region were then conducted to separately determine the double layer capacitance. Assumptions are made in our treatment of the electrochemically-derived ECSA data including equal conductivity of metals and oxides, negligible pseudo-capacitance, and consistent material specific capacitance, and are therefore suggested in benchmark methods to be accurate to within an order of magnitude of the true ECSA^53^. Due to low substrate coverage, we corrected the ECSA based on the double layer capacitance of the substrate with no electrodeposited material. As an added measure, we also approximated the ECSA using SEM and incorporated each value in the final ECSA determination. Linear sweep voltammograms were then conducted at 10 mV/s from the equilibrium potential for HER (0 V vs. RHE) or OER (1.23 V vs. RHE) to determine catalytic activity. The resulting voltammograms were corrected for pH and reference electrode by: *E*_RHE_ = *E*_Ag/AgCl_ + 0.198 + pH(0.059). Each system was evaluated three times to generate statistics on overpotential, Tafel slope, and mass activity, which can be found in the Supplementary Information.

### Source data

The source data for Figs. 1a and 4a, b are available as source data files.

## Supplementary information

Supporting Information

Source Data

## References

[1] Liu G (2013). Nanostructured high-strength molybdenum alloys with unprecedented tensile ductility. Nat. Mater..

[2] Stamenkovic VR (2007). Trends in electrocatalysis on extended and nanoscale Pt-bimetallic alloy surfaces. Nat. Mater..

[3] Yeh JW (2004). Nanostructured high-entropy alloys with multiple principal elements: novel alloy design concepts and outcomes. Adv. Eng. Mater..

[4] Cantor B, Chang ITH, Knight P, Vincent AJB (2004). Microstructural development in equiatomic multicomponent alloys. Mater. Sci. Eng. A.

[5] Guo S, Liu CT (2011). Phase stability in high entropy alloys: formation of solid-solution phase or amorphous phase. Proc. Nat. Sci. Mater..

[6] Gludovatz B (2014). A fracture-resistant high-entropy alloy for cryogenic applications. Science.

[7] Otto F (2013). The influences of temperature and microstructure on the tensile properties of a CoCrFeMnNi high-entropy alloy. Acta Mater..

[8] Wang YP, Li BS, Ren MX, Yang C, Fu HZ (2008). Microstructure and compressive properties of AlCrFeCoNi high entropy alloy. Mater. Sci. Eng. A.

[9] Zhang W, Liaw PK, Zhang Y (2018). Science and technology in high-entropy alloys. Sci. China Mater..

[10] Bligaard T, Nørskov JK (2007). Ligand effects in heterogeneous catalysis and electrochemistry. Electrochim. Acta.

[11] Kitchin JR, Nørskov JK, Barteau MA, Chen JG (2004). Role of strain and ligand effects in the modification of the electronic and chemical properties of bimetallic surfaces. Phys. Rev. Lett..

[12] Tsai MH, Yeh JW (2014). High-entropy alloys: a critical review. Mater. Res. Lett..

[13] Gao, M. C., Yeh, J.-W., Liaw, P. K. & Zhang, Y. *High-entropy alloys*. (Springer International Publishing, Switzerland, 2016).

[14] Pickering EJ, Jones NG (2016). High-entropy alloys: a critical assessment of their founding principles and future prospects. Int. Mater. Rev..

[15] Senkov ON, Miracle DB, Chaput KJ, Couzinie JP (2018). Development and exploration of refractory high entropy alloys—a review. J. Mater. Res..

[16] Greer AL (2009). Metallic glasses…on the threshold. Mater. Today.

[17] Ding HY, Yao KF (2013). High entropy Ti20Zr20Cu20Ni20Be20 bulk metallic glass. J. Non-Cryst. Solids.

[18] Miracle DB, Senkov ON (2017). A critical review of high entropy alloys and related concepts. Acta Mater..

[19] Reier T, Oezaslan M, Strasser P (2012). Electrocatalytic oxygen evolution reaction (OER) on Ru, Ir, and Pt catalysts: a comparative study of nanoparticles and bulk materials. ACS Catal..

[20] Yao YG (2018). Carbothermal shock synthesis of high-entropy-alloy nanoparticles. Science.

[21] Meng M (2014). Improved plasticity of bulk metallic glasses by electrodeposition. Mater. Sci. Eng. A.

[22] Peulon S, Lincot D (1996). Cathodic electrodeposition from aqueous solution of dense or open-structured zinc oxide films. Adv. Mater..

[23] Glasscott MW, Pendergast AD, Dick JE (2018). A universal platform for the electrodeposition of ligand-free metal nanoparticles from a water-in-oil emulsion system. ACS Appl. Nano Mater..

[24] Glasscott MW, Dick JE (2018). Direct electrochemical observation of single platinum cluster electrocatalysis on ultramicroelectrodes. Anal. Chem..

[25] Glasscott MW, Pendergast AD, Choudhury MH, Dick JE (2019). Advanced characterization techniques for evaluating porosity, nanopore tortuosity, and electrical connectivity at the single-nanoparticle level. ACS Appl. Nano Mater..

[26] Glasscott MW, Dick JE (2019). Fine-tuning porosity and time-resolved observation of the nucleation and growth of single platinum nanoparticles. ACS Nano.

[27] Dick JE, Lebegue E, Strawsine LM, Bard AJ (2016). Millisecond coulometry via zeptoliter droplet collisions on an ultramicroelectrode. Electroanalysis.

[28] Dick JE, Renault C, Kim BK, Bard AJ (2014). Simultaneous detection of single attoliter droplet collisions by electrochemical and electrogenerated chemiluminescent responses. Angew. Chem. Int. Ed..

[29] McEnaney JM (2014). Electrocatalytic hydrogen evolution using amorphous tungsten phosphide nanoparticles. Chem. Commun..

[30] Delmer O, Balaya P, Kienle L, Maier J (2008). Enhanced potential of amorphous electrode materials: case study of RuO_2_. Adv. Mater..

[31] Friel JJ, Lyman CE (2006). Tutorial review: x-ray mapping in electron-beam instruments. Microsc. Microanal..

[32] Healy JP, Pletcher D, Goodenough M (1992). The chemistry of the additives in an acid copper electroplating bath: part I. Polyethylene glycol and chloride ion. J. Electroanal. Chem..

[33] Chen PC (2016). Polyelemental nanoparticle libraries. Science.

[34] Zaveta K, Springmann B, Schneider J (1984). Magnetic aftereffects in amorphous Co- and CoNi-based alloys after field annealing. J. Magn. Magn. Mater..

[35] Li H (2017). Amorphous nickel-cobalt complexes hybridized with 1T-phase molybdenum disulfide via hydrazine-induced phase transformation for water splitting. Nat. Commun..

[36] Arroyo-Currás N, Bard AJ (2015). Iridium oxidation as observed by surface interrogation scanning electrochemical microscopy. J. Phys. Chem. C.

[37] Ahn HS, Bard AJ (2016). Surface interrogation scanning electrochemical microscopy of Ni1–xFexOOH (0 < x < 0.27) oxygen evolving catalyst: kinetics of the “fast” iron sites. J. Am. Chem. Soc..

[38] Huang EW (2015). A study of lattice elasticity from low entropy metals to medium and high entropy alloys. Scr. Mater..

[39] El-Seidy AM, Eissa AA (2015). The effect manganese concentration on the corrosion resistance and physical properties of Zn-Ni-Mn alloy films produced by electrodeposition. Int. J. Electrochem. Sci..

[40] Zangari G (2015). Electrodeposition of alloys and compounds in the era of microelectronics and energy conversion technology. Coatings.

[41] Sheng W, Gasteiger HA, Shao-Horn Y (2010). Hydrogen oxidation and evolution reaction kinetics on platinum: acid vs alkaline electrolytes. J. Electrochem. Soc..

[42] Jiao F, Frei H (2009). Nanostructured cobalt oxide clusters in mesoporous silica as efficient oxygen-evolving catalysts. Angew. Chem. Int. Ed..

[43] Gong L, Chng XYE, Du Y, Xi S, Yeo BS (2018). Enhanced catalysis of the electrochemical oxygen evolution reaction by iron(III) ions adsorbed on amorphous cobalt oxide. ACS Catal..

[44] Godwin IJ, Lyons MEG (2013). Enhanced oxygen evolution at hydrous nickel oxide electrodes via electrochemical ageing in alkaline solution. Electrochem. Commun..

[45] Burke MS, Enman LJ, Batchellor AS, Zou S, Boettcher SW (2015). Oxygen evolution reaction electrocatalysis on transition metal oxides and (oxy)hydroxides: activity trends and design principles. Chem. Mater..

[46] Abreu-Sepulveda MA (2016). The influence of Fe substitution in lanthanum calcium cobalt oxide on the oxygen evolution reaction in alkaline media. J. Electrochem. Soc..

[47] Sharma AS, Yadav S, Biswas K, Basu B (2018). High-entropy alloys and metallic nanocomposites: processing challenges, microstructure development and property enhancement. Mat. Sci. Eng. R.

[48] Goldsmith ZK (2017). Characterization of NiFe oxyhydroxide electrocatalysts by integrated electronic structure calculations and spectroelectrochemistry. Proc. Natl Acad. Sci. USA.

[49] McCrory CCL, Jung SH, Peters JC, Jaramillo TF (2013). Benchmarking heterogeneous electrocatalysts for the oxygen evolution reaction. J. Am. Chem. Soc..

[50] Chen GF (2016). Efficient and stable bifunctional electrocatalysts Ni/NixMy (M = P, S) for overall water splitting. Adv. Funct. Mater..

[51] Jin W, Du H, Zheng S, Xu H, Zhang Y (2010). Comparison of the oxygen reduction reaction between NaOH and KOH solutions on a Pt electrode: the electrolyte-dependent effect. J. Phys. Chem. B.

[52] Zeng K, Zhang D (2010). Recent progress in alkaline water electrolysis for hydrogen production and applications. Prog. Energy Combust. Sci..

[53] McCrory CCL (2015). Benchmarking hydrogen evolving reaction and oxygen evolving reaction electrocatalysts for solar water splitting devices. J. Am. Chem. Soc..

[54] Xiao H, Shin H, Goddard WA (2018). Synergy between Fe and Ni in the optimal performance of (Ni,Fe)OOH catalysts for the oxygen evolution reaction. Proc. Natl Acad. Sci. USA.

[55] Barforoush JM, Seuferling TE, Jantz DT, Song KR, Leonard KC (2018). Insights into the active electrocatalytic areas of layered double hydroxide and amorphous nickel–iron oxide oxygen evolution electrocatalysts. ACS Appl Energy Mater..

[56] Pendergast AD, Glasscott MW, Renault C, Dick JE (2019). One-step electrodeposition of ligand-free PdPt alloy nanoparticles from water droplets: Controlling size, coverage, and elemental stoichiometry. Electrochem. Commun..

[57] Jeun YE, Baek B, Lee MW, Ahn HS (2018). Surfactant-free electrochemical synthesis of metallic nanoparticles via stochastic collisions of aqueous nanodroplet reactors. Chem. Commun..

[58] Serrà A, Vallés E (2018). Microemulsion-based one-step electrochemical fabrication of mesoporous catalysts. Catalysts.

