# Publisher Correction: Electrosynthesis of high-entropy metallic glass nanoparticles for designer, multi-functional electrocatalysis

**DOI:** 10.1038/s41467-019-11219-4

**Published:** 2019-07-10

**Authors:** Matthew W. Glasscott, Andrew D. Pendergast, Sondrica Goines, Anthony R. Bishop, Andy T. Hoang, Christophe Renault, Jeffrey E. Dick

**Affiliations:** 1 Department of Chemistry, The University of North Carolina at Chapel Hill, Chapel Hill, NC 27599-3290 USA; 2 Laboratoire de Physique de la Matière Condensée, Ecole Polytechnique, CNRS, IP Paris, 91128 Palaiseau, France; 3 Lineberger Comprehensive Cancer Center, School of Medicine, The University of North Carolina at Chapel Hill, Chapel Hill, NC 27599-3290 USA

**Keywords:** Electrocatalysis, Renewable energy, Metals and alloys

Correction to: *Nature Communications* 10.1038/s41467-019-10303-z, published online 14 June 2019.

The original version of this Article contained an error in Fig. 4a, in which an extra label ‘H_2_’ was inadvertently added. This has been corrected in both the PDF and HTML versions of the Article.

The correct version is:

It replaces the incorrect version:

