# Supplementary material for: Electrosynthesis of high-entropy metallic glass nanoparticles for designer, multi-functional electrocatalysis
Source: Nat Commun. 2019 Jun 14;10:2650. doi: 10.1038/s41467-019-10303-z (PMC6570760; doi:10.1038/s41467-019-10303-z)
Supplement: Supplementary file 1 — Supporting Information [file 41467_2019_10303_MOESM1_ESM.pdf]

Supplementary Information:

Electrosynthesis of High-Entropy Metallic Glass Nanoparticles for Designer, Multi-Functional Electrocatalysis

Glasscott *et al.*

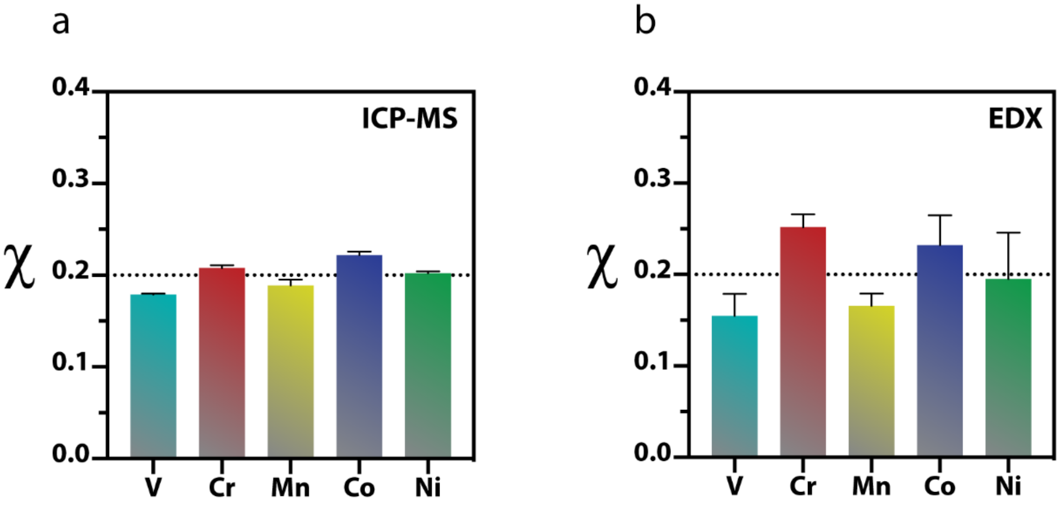

Supplementary Figure 1 | ICP-MS of Quinary CoCrMnNiV HEMG-NPs. Elemental stoichiometry of individual metal components in CoCrMnNiV HEMG-NPs from (a) ICP-MS and (b) EDX demonstrating control of elemental ratios from droplets loaded with equimolar metal precursor.

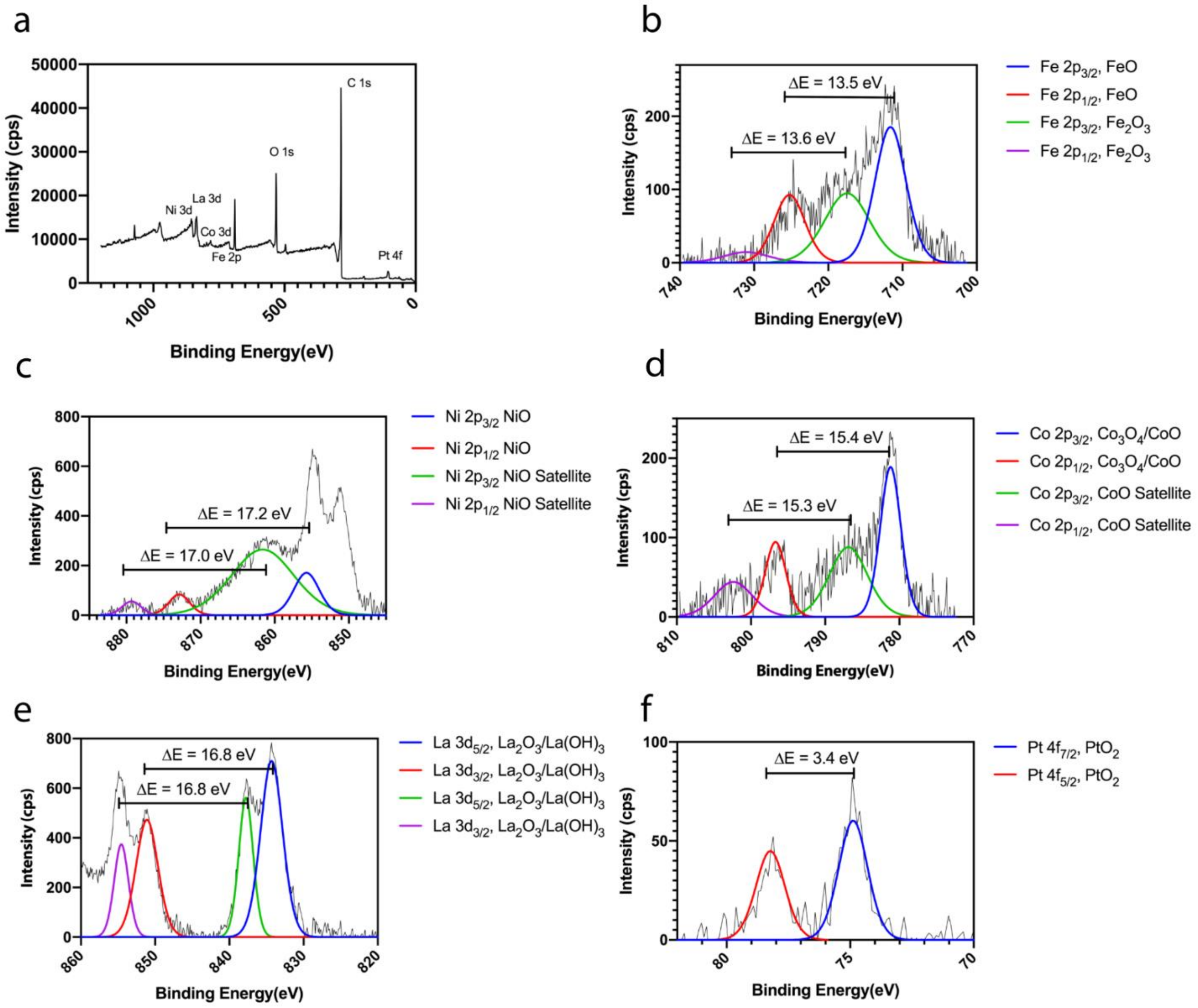

Supplementary Figure 2 | XPS of CoFeLaNiPt HEMG-NP (a) XPS survey scan for a high coverage CoFeMnNiPt is presented demonstrating characteristic carbon and oxygen regions in addition to distinct XPS peaks corresponding to individual metallic species. (b) Fe 2p XPS region demonstrates mixed FeO and Fe_2O_3 species at $2p_{3/2}$ binding energies of 711.1 eV (ΔE = 13.5 eV) and 716.8 eV (ΔE = 13.6 eV) respectively. (c) Co 2p XPS region with characteristic mixed CoO and Co_3O_4 XPS peaks at a binding energy of 780.8 eV (ΔE = 15.4 eV) with a characteristic satellite peak at a binding energy of 786.1 eV (ΔE = 15.3 eV). (d) Ni 2p XPS region with the presence of a peak corresponding to NiO at a binding energy of 855.0 eV (ΔE = 17.2 eV) and a satellite peak at binding energy 861.6 eV (ΔE = 17.0 eV). (e) La 3d XPS region with two peaks at binding energies of 833.9 eV (ΔE = 17.0 eV) and 837.5 eV (ΔE = 16.8 eV) potentially corresponding to convoluted La_2O_3 and $La(OH)_3$ XPS signatures. (f) Pt 4f XPS region with the presence of a distinct peak at a binding energy of 74.7 eV (ΔE = 3.4 eV) corresponding to the presence of PtO_2.

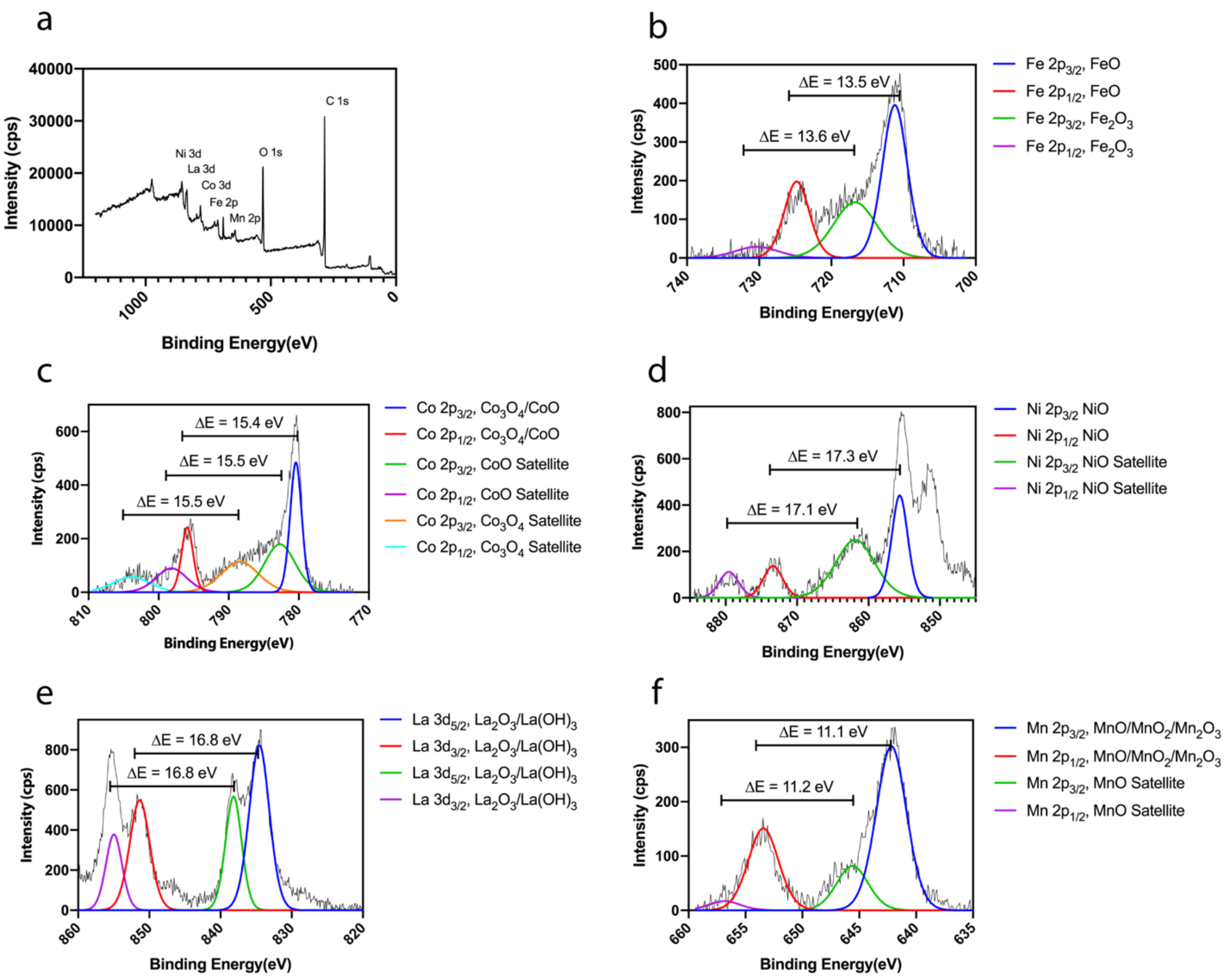

Supplementary Figure 3 | XPS of CoFeLaMnNi HEMG-NP (a) XPS survey scan for a high coverage CoFeLaMnNi is presented demonstrating characteristic carbon and oxygen regions in addition to distinct XPS peaks corresponding to individual metallic species. (b) Fe 2p XPS region demonstrates mixed FeO and Fe_2O_3 species at $2p_{3/2}$ binding energies of 710.7 eV (ΔE = 13.5 eV) and 716.0 eV (ΔE = 13.6 eV) respectively. (c) Co 2p XPS region with characteristic mixed CoO and Co_3O_4 XPS peaks at a binding energy of 780.2 eV (ΔE = 15.4 eV) with a characteristic satellite peak at a binding energy of 787.7 eV (ΔE = 15.5 eV) and a distinct CoO satellite peak at a binding energy of 782.0 eV (ΔE = 15.5 eV). (d) Ni 2p XPS region with the presence of a peak corresponding to NiO at a binding energy of 855.3 eV (ΔE = 17.3 eV) and a satellite peak at binding energy 861.8 eV (ΔE = 17.1 eV). (e) La 3d XPS region with two peaks at binding energies of 834.2 eV (ΔE = 16.8 eV) and 837.8 eV (ΔE = 16.8 eV) potentially corresponding to convoluted La_2O_3 and $La(OH)_3$ XPS signatures. (f) Mn 2p XPS region with the presence of a mixed MnO, MnO_2, and Mn_2O_3 peak at a binding energy of 641.8 eV (ΔE = 11.1 eV) with a satellite XPS peak at a binding energy of 645.1 eV (ΔE = 11.2 eV).

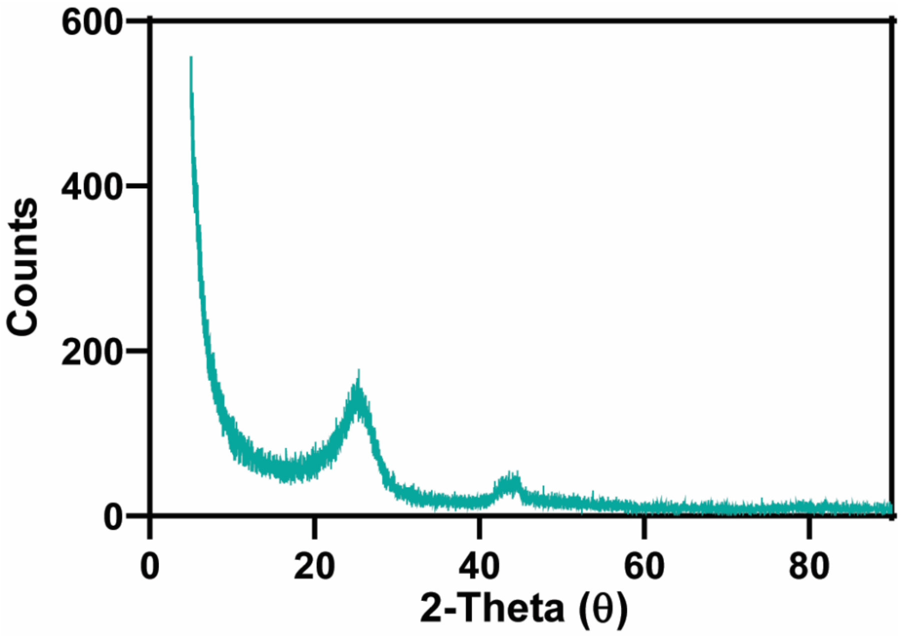

Supplementary Figure 4 | XRD of as-deposited HEMG-NPs. X-ray diffraction spectra of CoFeLaMnNi HEMG-NP high coverage sample on amorphous glassy carbon substrate electrode in a 2-Theta grazing angle orientation indicating characteristic glassy carbon broad amorphous peaks and no significant sharp peaks corresponding to metallic crystal facets or crystalline alloys.

\

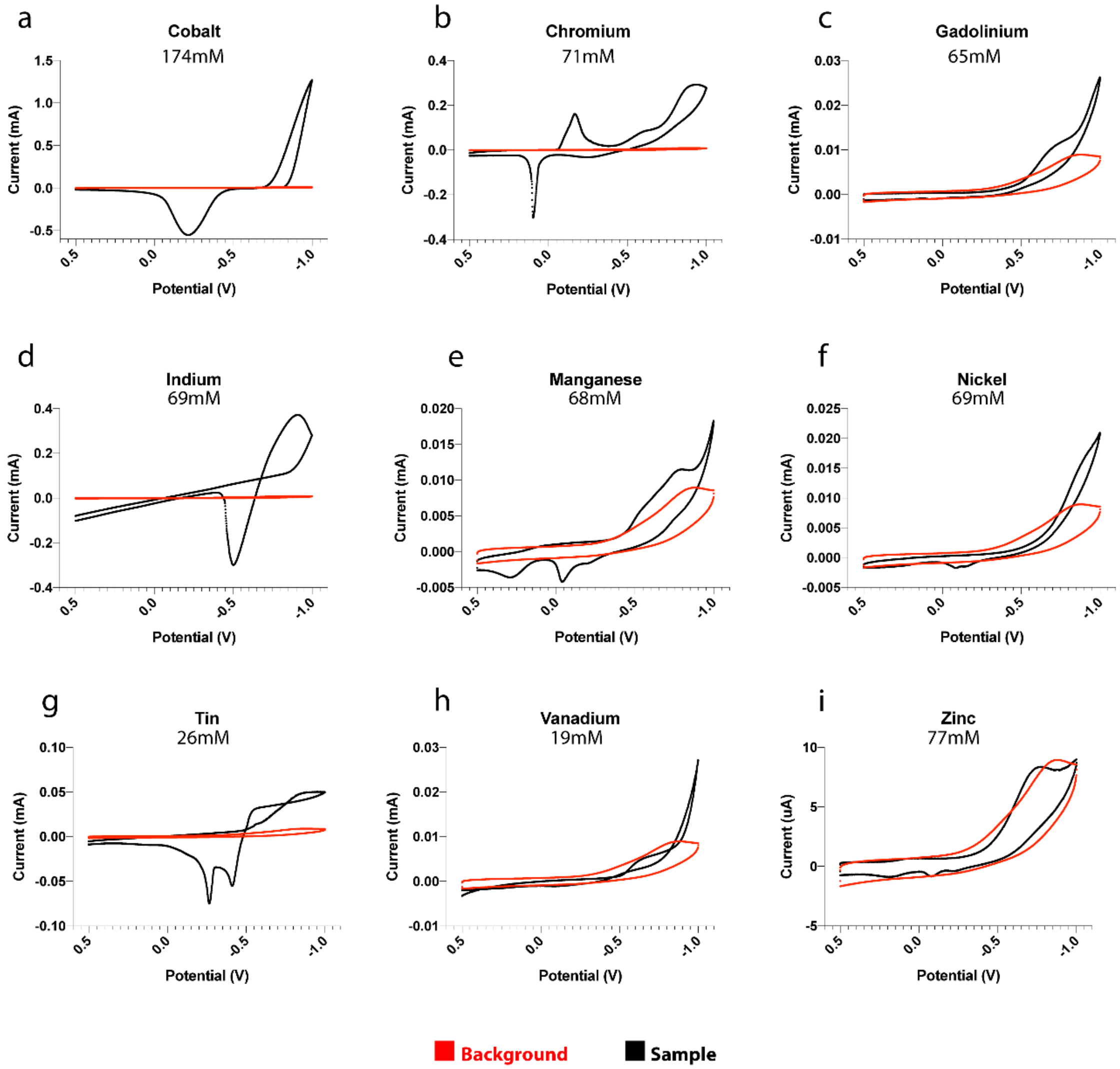

Supplementary Figure 5 | Cyclic voltammetry traces of individual metal chlorides. CV scans of metal chloride precursor on a 3 mm diameter HOPG electrode for electrodeposition with 250 mM KCl and 137 mM phosphate buffered saline (1X PBS) with a glassy carbon rod counter electrode and a double-junction Ag/AgCl reference electrode. Cathodic scans from 0.5 V *vs.* Ag/AgCl reveal nucleation behavior at varying onset potentials for (a) $CoCl_2$, (b) $CrCl_3$, (c) $GdCl_3$, (d) $InCl_3$, e) $MnCl_2$, (f) $NiCl_2$, (g) $SnCl_2$, (h) VCl_3, (i) $ZnCl_2$. Under these experimental conditions, metals contained in droplets may initially electrodeposit on the carbon substrate, and eventually a newly formed metal phase, making a kinetic evaluation of electrodeposition difficult. This evaluation is difficult since the nucleation and growth of metals is an inner-sphere process, and the reduction of each metal species on corresponding dissimilar metal electrodes has not been studied in detail. To further this complication, the potential at which single atoms can be oxidized is different from bulk, polycrystalline metal, implying that differences in heterogeneous kinetics may manifest from very small agglomerates of metal atoms. Finally, the stabilization of deposited metal atoms on the growing amorphous structure is not well understood.

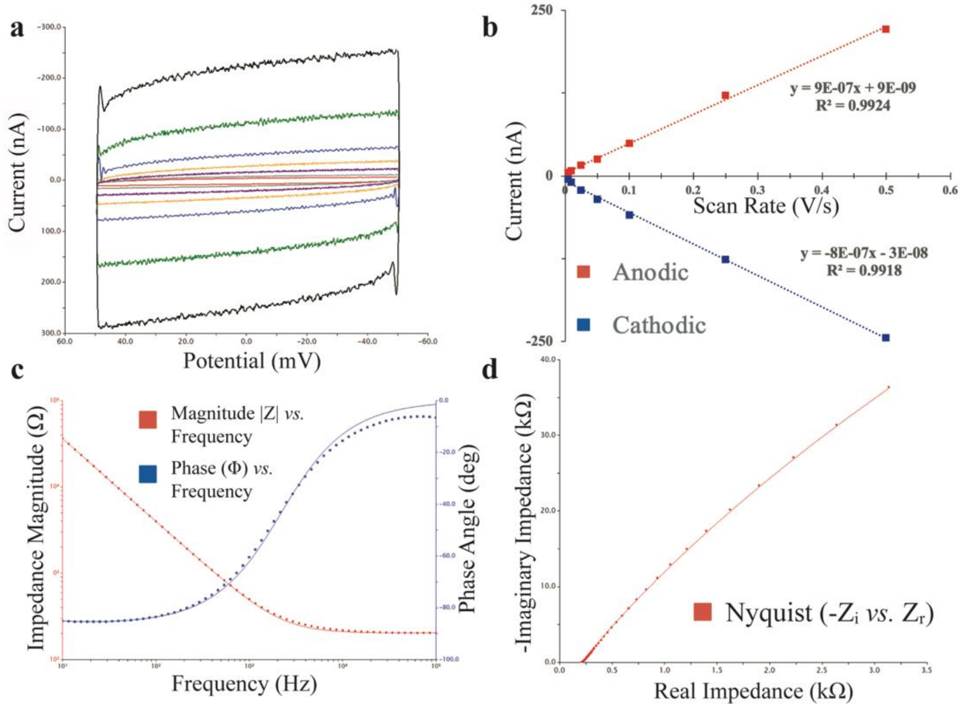

Supplementary Figure 6 | ECSA of electrocatalytic materials: Co HER. Determination of electrochemically active surface area of a HOPG electrode decorated with Co NPs as described in the Materials and Methods section. (a) Cyclic voltammetry of Co NP decorated electrode at 5, 10, 25, 50, 100, 250, and 500 mV/s in a potential range of -50 mV to 50 mV *vs.* Ag/AgCl for ECSA determination based on the double layer capacitance in a non-faradaic region. (b) Linear regression determination of ECSA by fitting of non-faradaic current to obtain the double layer capacitance. (c) Bode magnitude and phase plots of electrochemical impedance spectroscopy. (d) Nyquist plot of electrochemical impedance spectroscopy results fit with a Powell method over 500 iterations with parametric weighting and a $X^2 = 0.07328$.

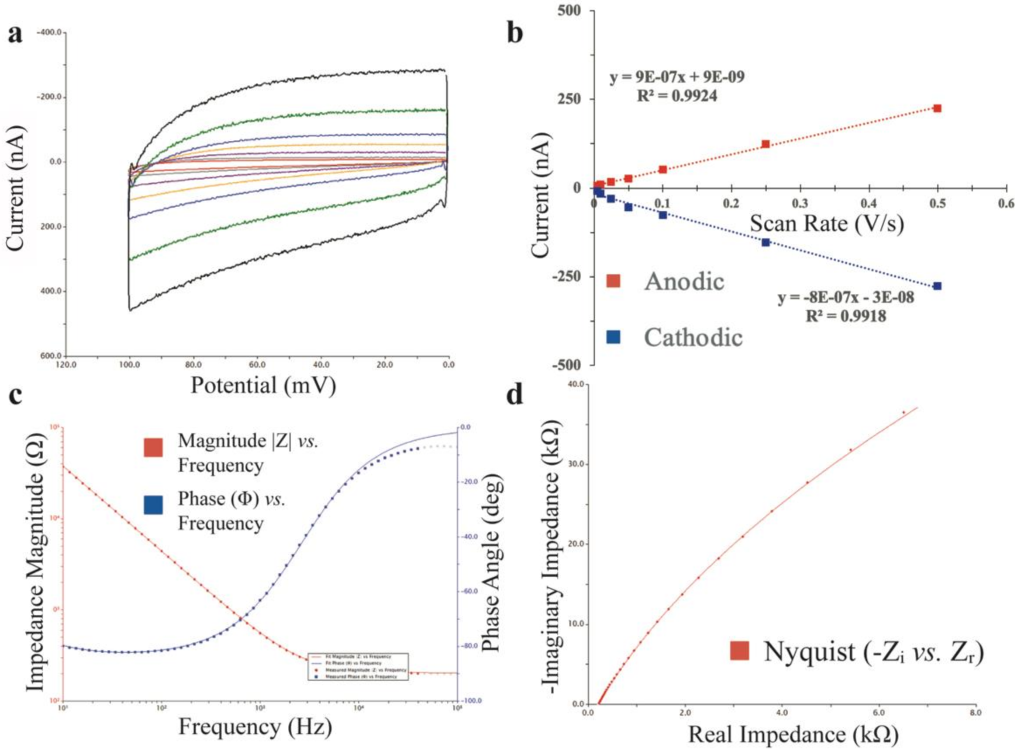

Supplementary Figure 7 | ECSA of electrocatalytic materials: Co OER. Determination of electrochemically active surface area of a glassy carbon rotating disk electrode decorated with Co NPs as described in the Materials and Methods section. (a) Cyclic voltammetry of Co NP decorated electrode at 5, 10, 25, 50, 100, 250, and 500 mV/s in a potential range of -50 mV to 50 mV *vs.* Ag/AgCl for ECSA determination based on the double layer capacitance in a non-faradaic region. (b) Linear regression determination of ECSA by fitting of non-faradaic current to obtain the double layer capacitance. (c) Bode magnitude and phase plots of electrochemical impedance spectroscopy. (d) Nyquist plot of electrochemical impedance spectroscopy results fit with a Powell method over 500 iterations with parametric weighting and a $X^2 = 0.07328$.

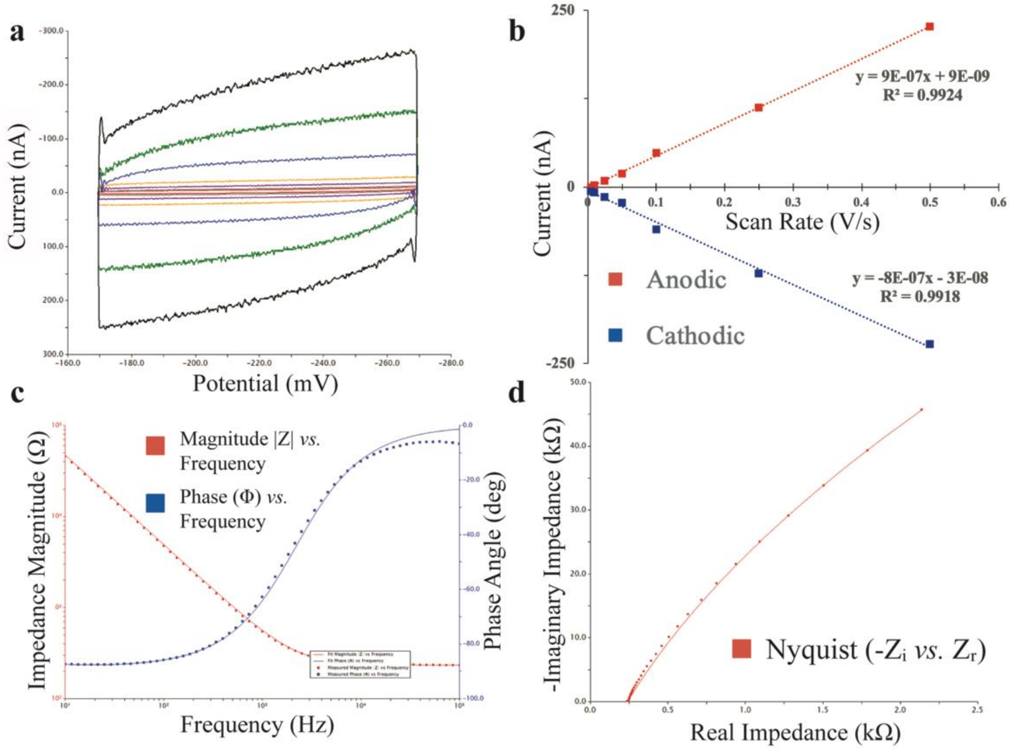

Supplementary Figure 8 | ECSA of electrocatalytic materials: Fe HER. Determination of electrochemically active surface area of a glassy carbon rotating disk electrode decorated with Fe NPs as described in the Materials and Methods section. (a) Cyclic voltammetry of Fe NP decorated electrode at 5, 10, 25, 50, 100, 250, and 500 mV/s in a potential range of -50 mV to 50 mV *vs.* Ag/AgCl for ECSA determination based on the double layer capacitance in a non-faradaic region. (b) Linear regression determination of ECSA by fitting of non-faradaic current to obtain the double layer capacitance. (c) Bode magnitude and phase plots of electrochemical impedance spectroscopy. (d) Nyquist plot of electrochemical impedance spectroscopy results fit with a Powell method over 500 iterations with parametric weighting and a $X^2 = 0.07328$.

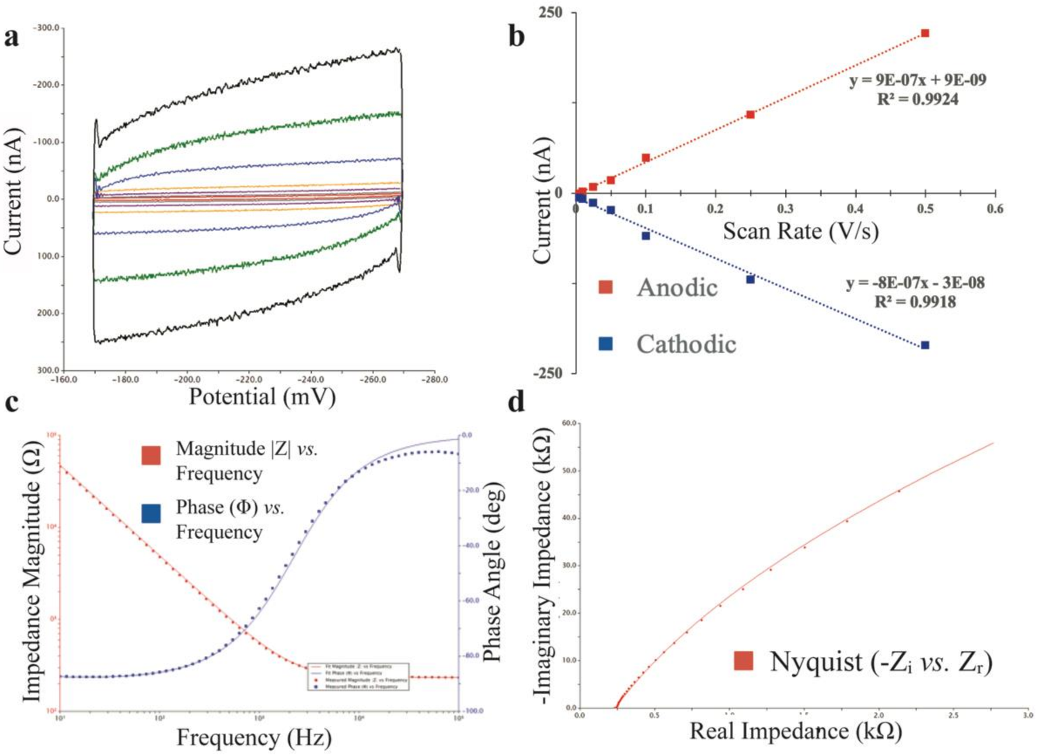

Supplementary Figure 9 | ECSA of electrocatalytic materials: Fe OER. Determination of electrochemically active surface area of a glassy carbon rotating disk electrode decorated with Fe NPs as described in the Materials and Methods section. (a) Cyclic voltammetry of Fe NP decorated electrode at 5, 10, 25, 50, 100, 250, and 500 mV/s in a potential range of -50 mV to 50 mV *vs.* Ag/AgCl for ECSA determination based on the double layer capacitance in a non-faradaic region. (b) Linear regression determination of ECSA by fitting of non-faradaic current to obtain the double layer capacitance. (c) Bode magnitude and phase plots of electrochemical impedance spectroscopy. (d) Nyquist plot of electrochemical impedance spectroscopy results fit with a Powell method over 500 iterations with parametric weighting and a $X^2 = 0.07328$.

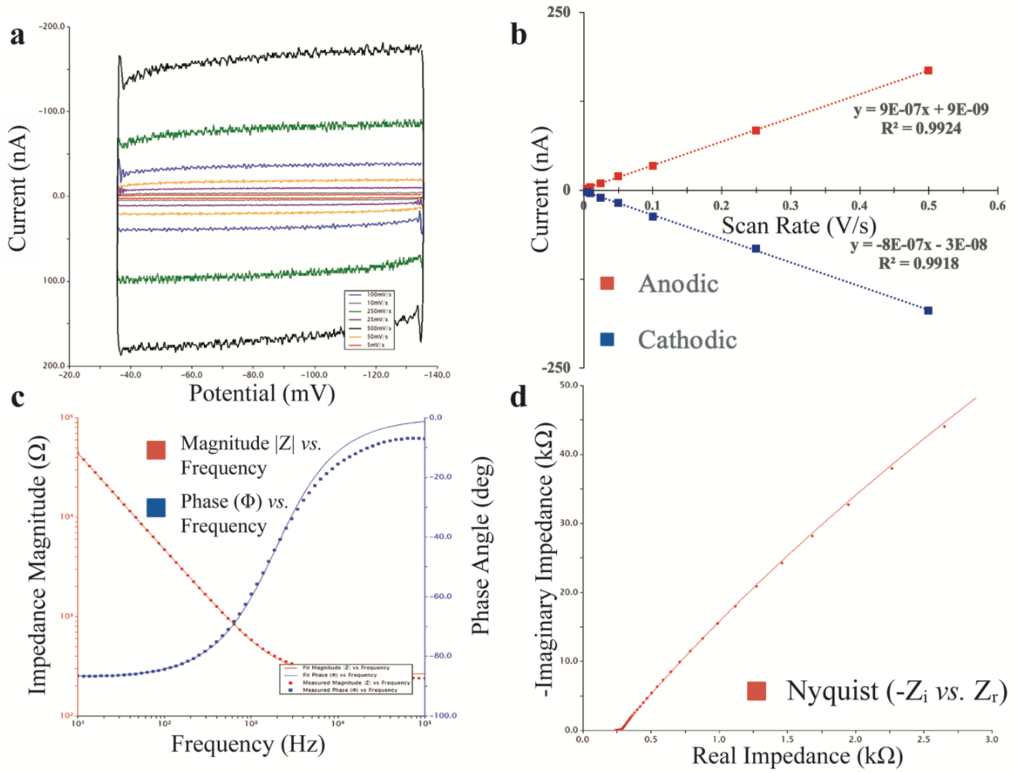

Supplementary Figure 10 | ECSA of electrocatalytic materials: La HER. Determination of electrochemically active surface area of a glassy carbon rotating disk electrode decorated with La NPs as described in the Materials and Methods section. (a) Cyclic voltammetry of La NP decorated electrode at 5, 10, 25, 50, 100, 250, and 500 mV/s in a potential range of -50 mV to 50 mV *vs.* Ag/AgCl for ECSA determination based on the double layer capacitance in a non-faradaic region. (b) Linear regression determination of ECSA by fitting of non-faradaic current to obtain the double layer capacitance. (c) Bode magnitude and phase plots of electrochemical impedance spectroscopy. (d) Nyquist plot of electrochemical impedance spectroscopy results fit with a Powell method over 500 iterations with parametric weighting and a $X^2 = 0.07328$.

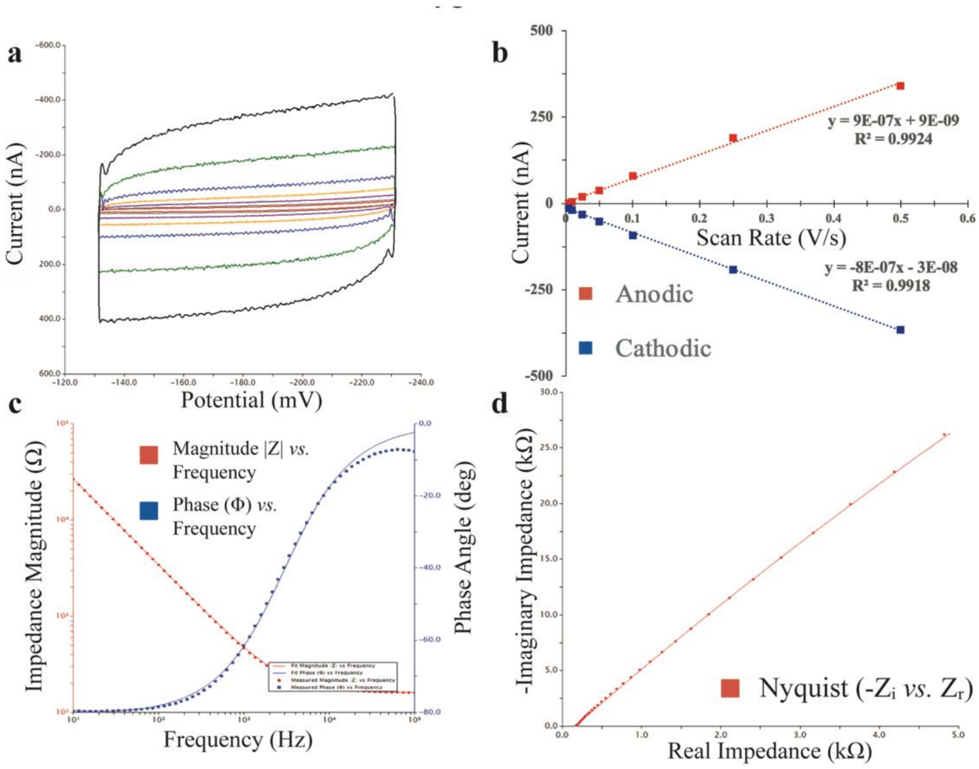

Supplementary Figure 11 | ECSA of electrocatalytic materials: La OER. Determination of electrochemically active surface area of a glassy carbon rotating disk electrode decorated with La NPs as described in the Materials and Methods section. (a) Cyclic voltammetry of La NP decorated electrode at 5, 10, 25, 50, 100, 250, and 500 mV/s in a potential range of -50 mV to 50 mV *vs.* Ag/AgCl for ECSA determination based on the double layer capacitance in a non-faradaic region. (b) Linear regression determination of ECSA by fitting of non-faradaic current to obtain the double layer capacitance. (c) Bode magnitude and phase plots of electrochemical impedance spectroscopy. (d) Nyquist plot of electrochemical impedance spectroscopy results fit with a Powell method over 500 iterations with parametric weighting and a $X^2 = 0.07328$.

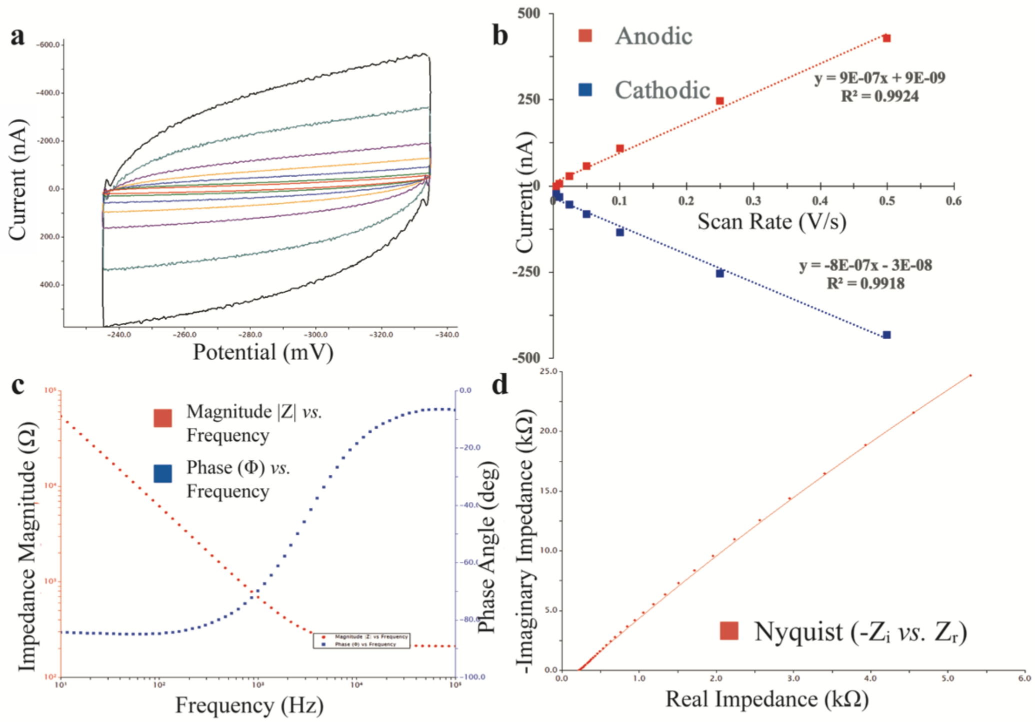

Supplementary Figure 12 | ECSA of electrocatalytic materials: Pt HER. Determination of electrochemically active surface area of a glassy carbon rotating disk electrode decorated with Pt NPs as described in the Materials and Methods section. (a) Cyclic voltammetry of Pt NP decorated electrode at 5, 10, 25, 50, 100, 250, and 500 mV/s in a potential range of -50 mV to 50 mV *vs.* Ag/AgCl for ECSA determination based on the double layer capacitance in a non-faradaic region. (b) Linear regression determination of ECSA by fitting of non-faradaic current to obtain the double layer capacitance. (c) Bode magnitude and phase plots of electrochemical impedance spectroscopy. (d) Nyquist plot of electrochemical impedance spectroscopy results fit with a Powell method over 500 iterations with parametric weighting and a X^2 = 0.07328.

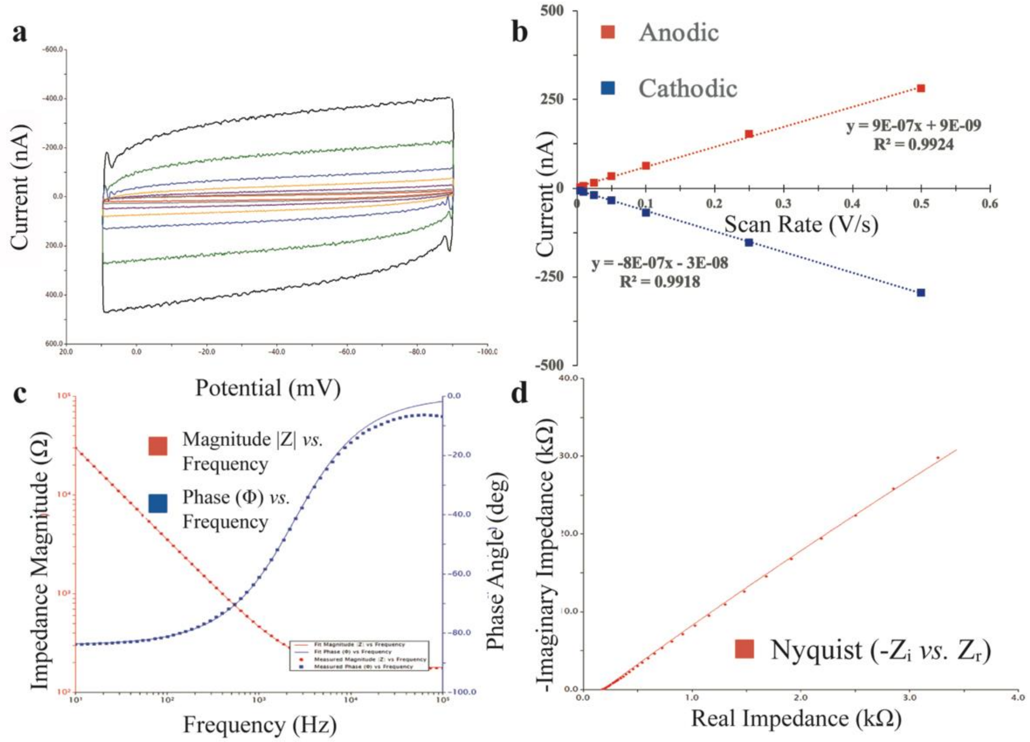

Supplementary Figure 13 | ECSA of electrocatalytic materials: Pt OER. Determination of electrochemically active surface area of a glassy carbon rotating disk electrode decorated with Pt NPs as described in the Materials and Methods section. (a) Cyclic voltammetry of Pt NP decorated electrode at 5, 10, 25, 50, 100, 250, and 500 mV/s in a potential range of -50 mV to 50 mV *vs.* Ag/AgCl for ECSA determination based on the double layer capacitance in a non-faradaic region. (b) Linear regression determination of ECSA by fitting of non-faradaic current to obtain the double layer capacitance. (c) Bode magnitude and phase plots of electrochemical impedance spectroscopy. (d) Nyquist plot of electrochemical impedance spectroscopy results fit with a Powell method over 500 iterations with parametric weighting and a X^2 = 0.07328.

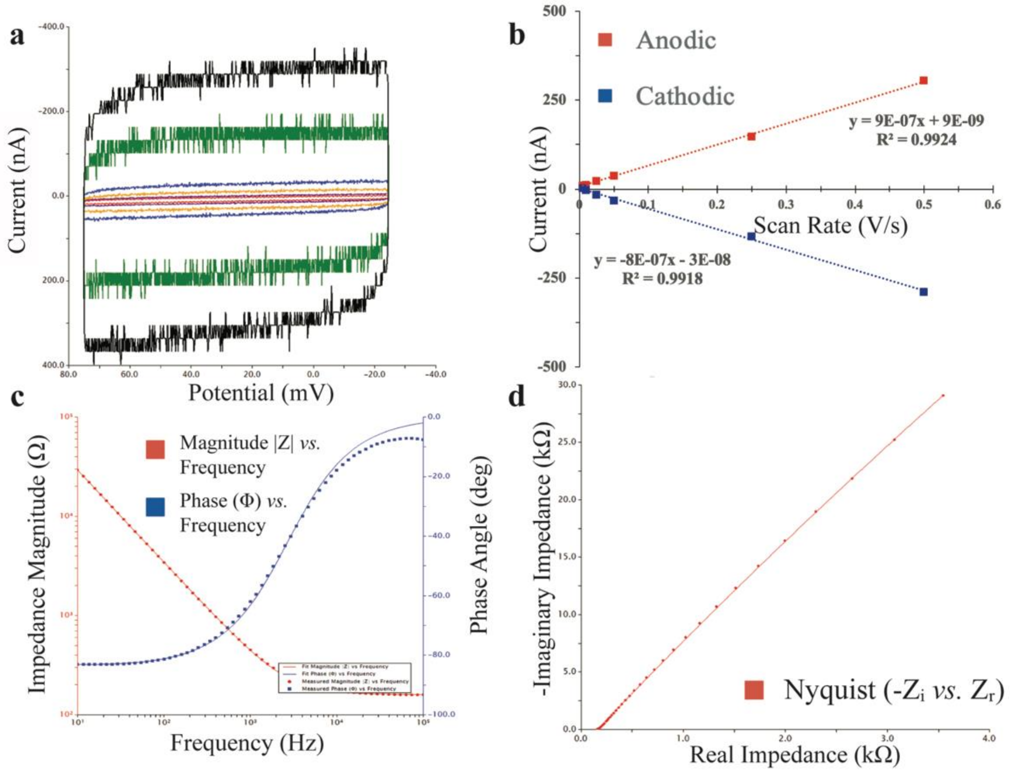

Supplementary Figure 14 | ECSA of electrocatalytic materials: Ni HER. Determination of electrochemically active surface area of a glassy carbon rotating disk electrode decorated with Ni NPs as described in the Materials and Methods section. (a) Cyclic voltammetry of Ni NP decorated electrode at 5, 10, 25, 50, 100, 250, and 500 mV/s in a potential range of -50 mV to 50 mV *vs.* Ag/AgCl for ECSA determination based on the double layer capacitance in a non-faradaic region. (b) Linear regression determination of ECSA by fitting of non-faradaic current to obtain the double layer capacitance. (c) Bode magnitude and phase plots of electrochemical impedance spectroscopy. (d) Nyquist plot of electrochemical impedance spectroscopy results fit with a Powell method over 500 iterations with parametric weighting and a X^2 = 0.07328.

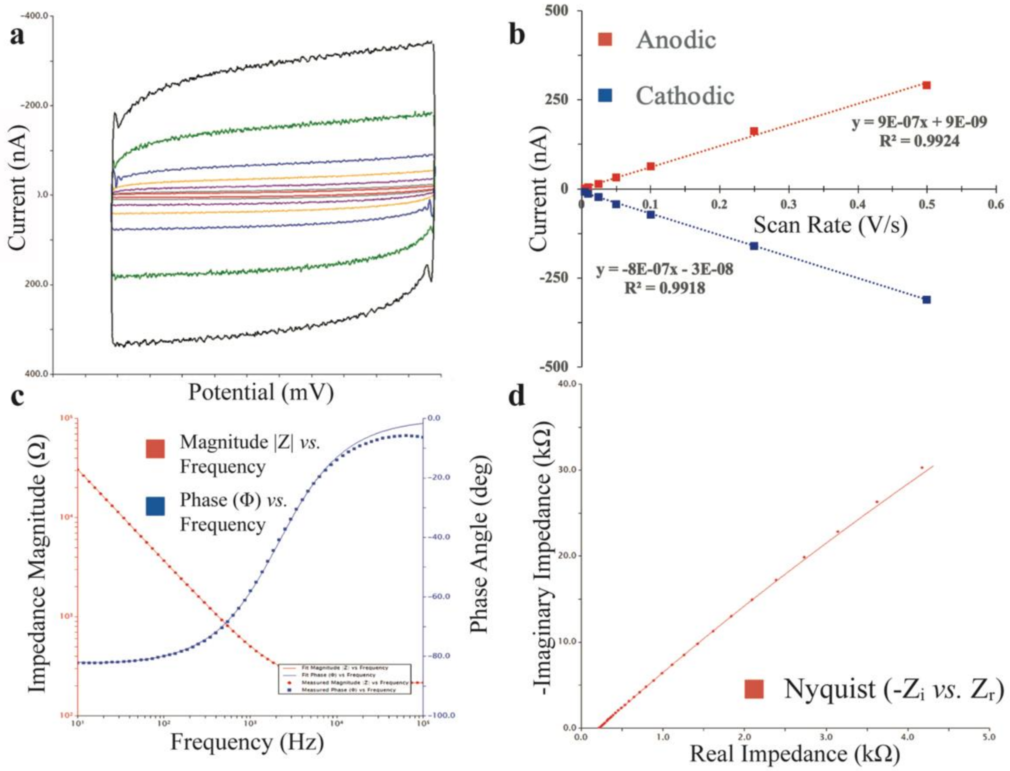

Supplementary Figure 15 | ECSA of electrocatalytic materials: Ni OER. Determination of electrochemically active surface area of a glassy carbon rotating disk electrode decorated with Ni NPs as described in the Materials and Methods section(a) Cyclic voltammetry of Ni NP decorated electrode at 5, 10, 25, 50, 100, 250, and 500 mV/s in a potential range of -50 mV to 50 mV *vs.* Ag/AgCl for ECSA determination based on the double layer capacitance in a non-faradaic region. (b) Linear regression determination of ECSA by fitting of non-faradaic current to obtain the double layer capacitance. (c) Bode magnitude and phase plots of electrochemical impedance spectroscopy. (d) Nyquist plot of electrochemical impedance spectroscopy results fit with a Powell method over 500 iterations with parametric weighting and a X^2 = 0.07328.

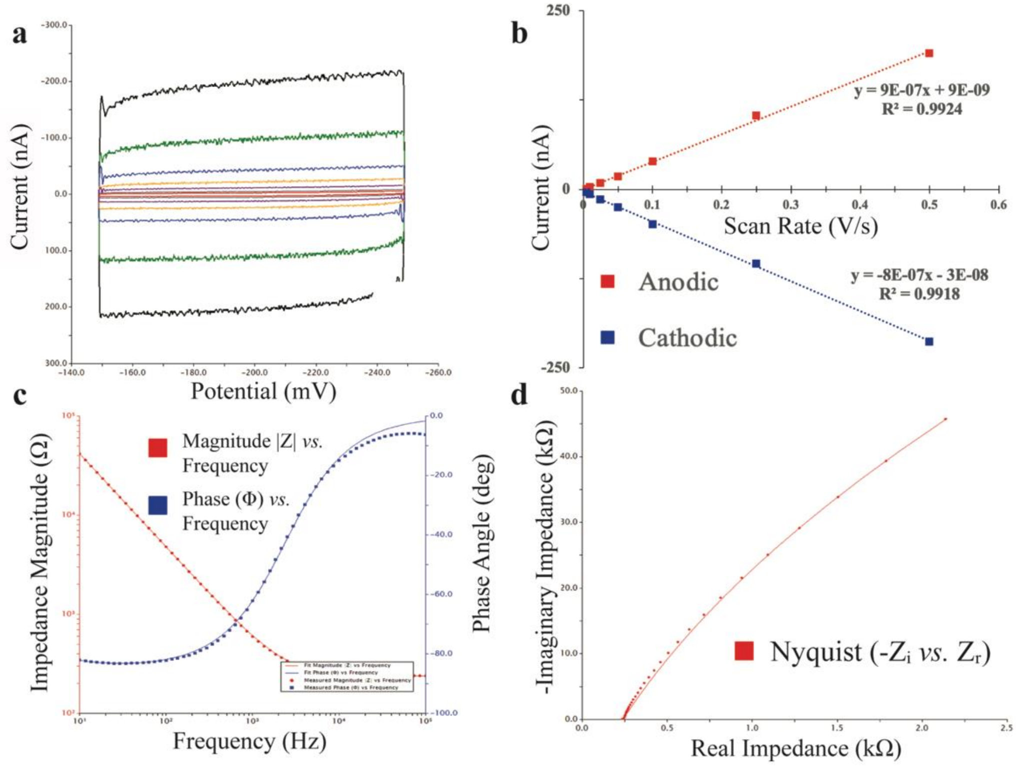

Supplementary Figure 16 | ECSA of electrocatalytic materials: HEMG CoFeLaNiPt HER. Determination of electrochemically active surface area of a glassy carbon rotating disk electrode decorated with HEMG-NPs as described in the Materials and Methods section. (a) Cyclic voltammetry of HEMG-NP decorated electrode at 5, 10, 25, 50, 100, 250, and 500 mV/s in a potential range of -50 mV to 50 mV *vs.* Ag/AgCl for ECSA determination based on the double layer capacitance in a non-faradaic region. (b) Linear regression determination of ECSA by fitting of non-faradaic current to obtain the double layer capacitance. (c) Bode magnitude and phase plots of electrochemical impedance spectroscopy. (d) Nyquist plot of electrochemical impedance spectroscopy results fit with a Powell method over 500 iterations with parametric weighting and a $X^2 = 0.07328$.

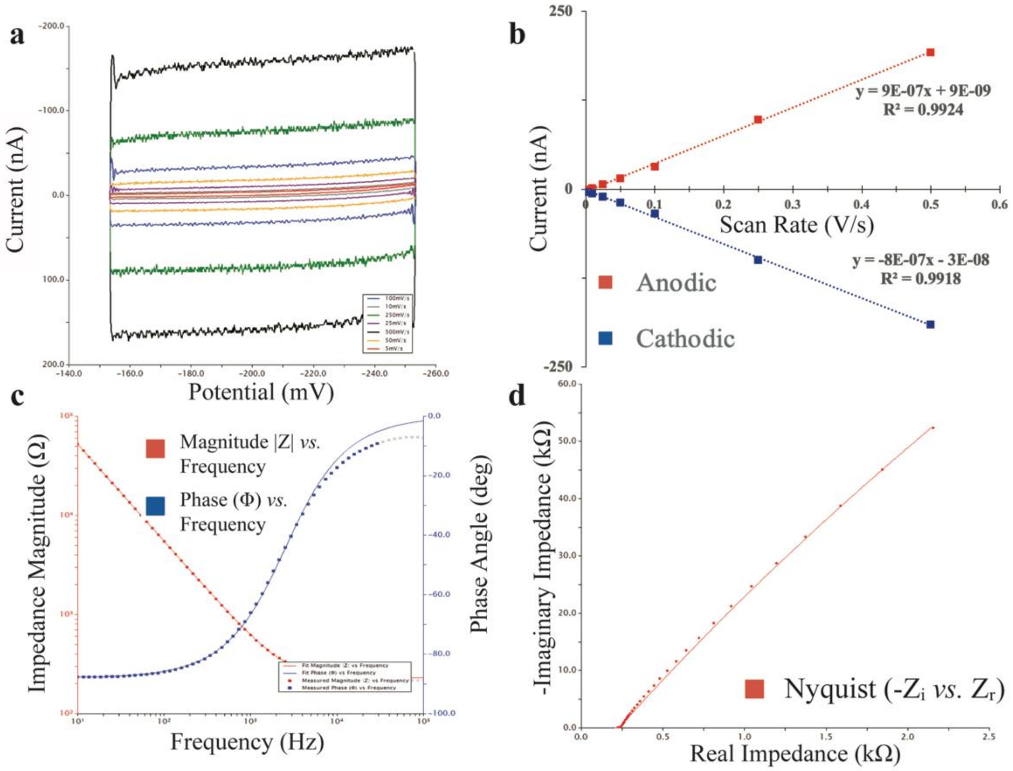

Supplementary Figure 17 | ECSA of electrocatalytic materials: HEMG CoFeLaNiPt HER. Determination of electrochemically active surface area of a glassy carbon rotating disk electrode decorated with HEMG-NPs as described in the Materials and Methods section. (a) Cyclic voltammetry of HEMG-NP decorated electrode at 5, 10, 25, 50, 100, 250, and 500 mV/s in a potential range of -50 mV to 50 mV *vs.* Ag/AgCl for ECSA determination based on the double layer capacitance in a non-faradaic region. (b) Linear regression determination of ECSA by fitting of non-faradaic current to obtain the double layer capacitance. (c) Bode magnitude and phase plots of electrochemical impedance spectroscopy. (d) Nyquist plot of electrochemical impedance spectroscopy results fit with a Powell method over 500 iterations with parametric weighting and a $X^2 = 0.07328$.

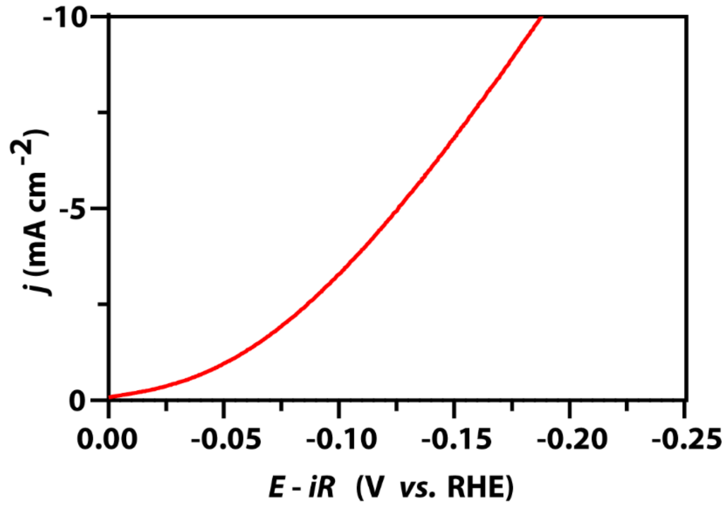

Supplementary Figure 18 | 1 mm Pt Disk Electrode HER in 0.1 M KOH. Linear sweep voltammogram showing the polarization of a 1 mm radius Pt disk electrode to drive the HER. A current density of 10 mA·cm^{-2} is reached at -201 mV vs. RHE, corresponding to an overpotential of 201 mV. The ECSA of the electrode was determined by EIS to be 0.0993 cm^{2} assuming 20 μF·cm^{-2} as the specific capacitance. A Ag/AgCl reference electrode separated from the cell by a salt bridge and a glassy carbon rod acted as the reference and counter electrode, respectively. Scan rate was 10 mV/s.

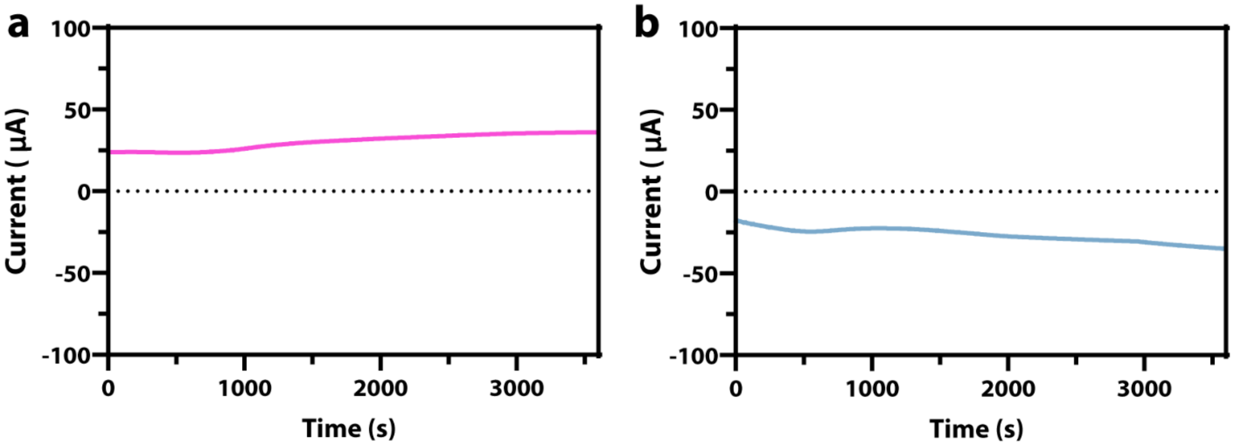

Supplementary Figure 19 | HEMG-NP stability for OER/HER. (a) Stability of the CoFeLaNiPt HEMG-NP electrocatalyst over 3600 seconds at an overpotential of 377 mV (corresponding to an initial current density j = 10 mA·cm^{-2}) for the OER at 25° C and under conditions of O_2 saturation revealing minimal drift in current density. (b) Stability of CoFeLaNiPt HEMG-NP electrocatalyst over 3600 seconds at an overpotential of 557 mV (corresponding to an initial current density j = -10 mA·cm^{-2}) for the HER at 25° C and under conditions of H_2 saturation revealing minimal drift in current density.

a

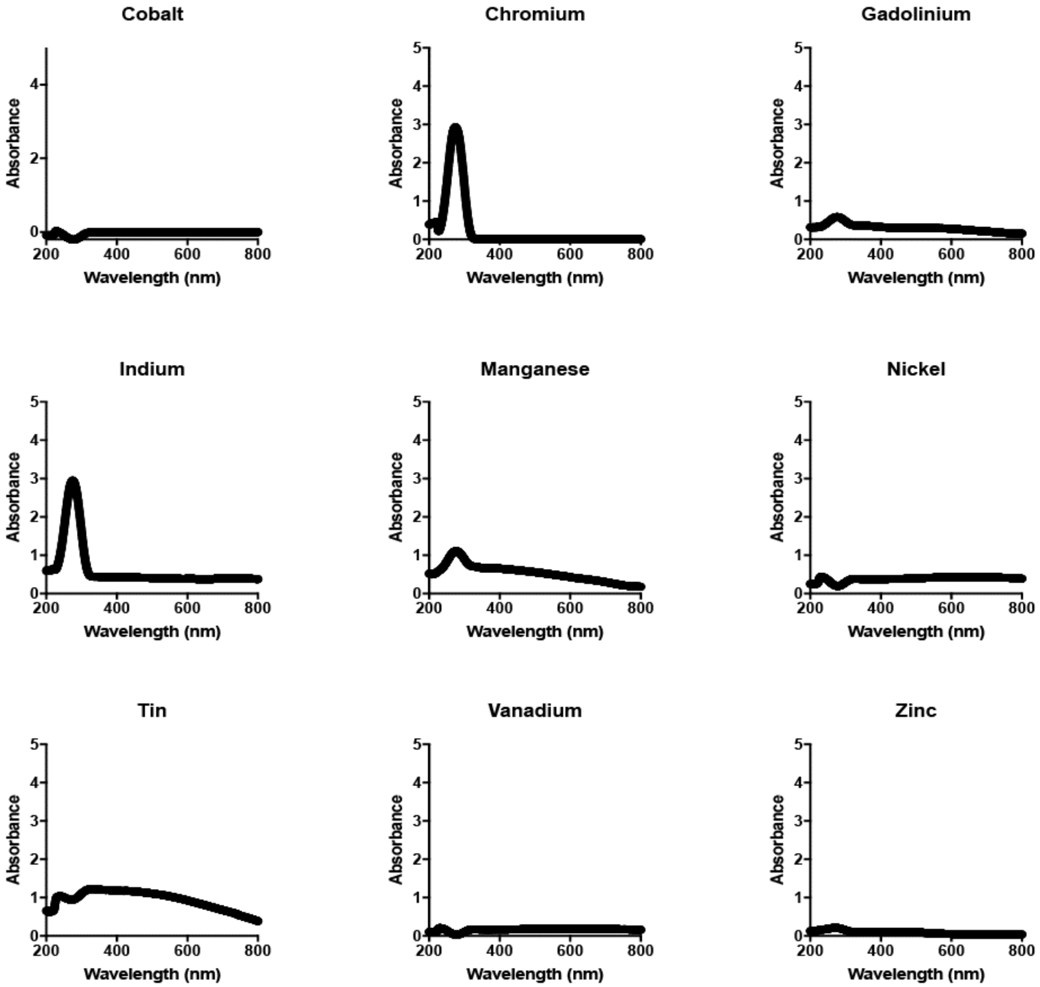

b

Metal	Cobalt	Chromium	Gadolinium	Indium	Manganese	Nickel	Tin	Vanadium	Zinc
Max Concentration µM	0.033	2.896	0.558	2.648	1.112	0.218	0.730	0.168	0.199

Supplementary Figure 20 | Determination of metal salt leakage into organic phase by UV-vis. Metal salt precursor leakage from the aqueous nanodroplet phase to the organic DCE phase was investigated by UV-vis spectroscopy. In brief, 10 µL of each individual metal salt precursor was mixed with 20 µL ultrapure water and suspended in a 5 mL DCE continuous phase with 0.1 M TBAP and an emulsion was prepared as outlined in the Materials and Methods section. Following sonication, the emulsion mixture was centrifuged in an Eppendorf Centrifuge 5804 R for 10 minutes at 4200 rpm to separate the aqueous and organic phases. A 2 mL aliquot of the DCE phase was extracted and absorbance spectra were collected with a JASCO V-650 Spectrophotometer in the 200-800 nm wavelength range. (a) UV-vis spectra of the DCE fraction containing $CoCl_2$, $CrCl_3$, $GdCl_3$, $InCl_3$, $MnCl_2$, $NiCl_2$, $SnCl_2$, VCl_3, and $ZnCl_2$. (b) Theoretical maximum metal salt concentrations in DCE continuous phase following emulsion/centrifugation procedure calculated from Beer-Lambert Law (path length = 1 cm), suggesting minimal metal salt precursor leakage in the micromolar regime.

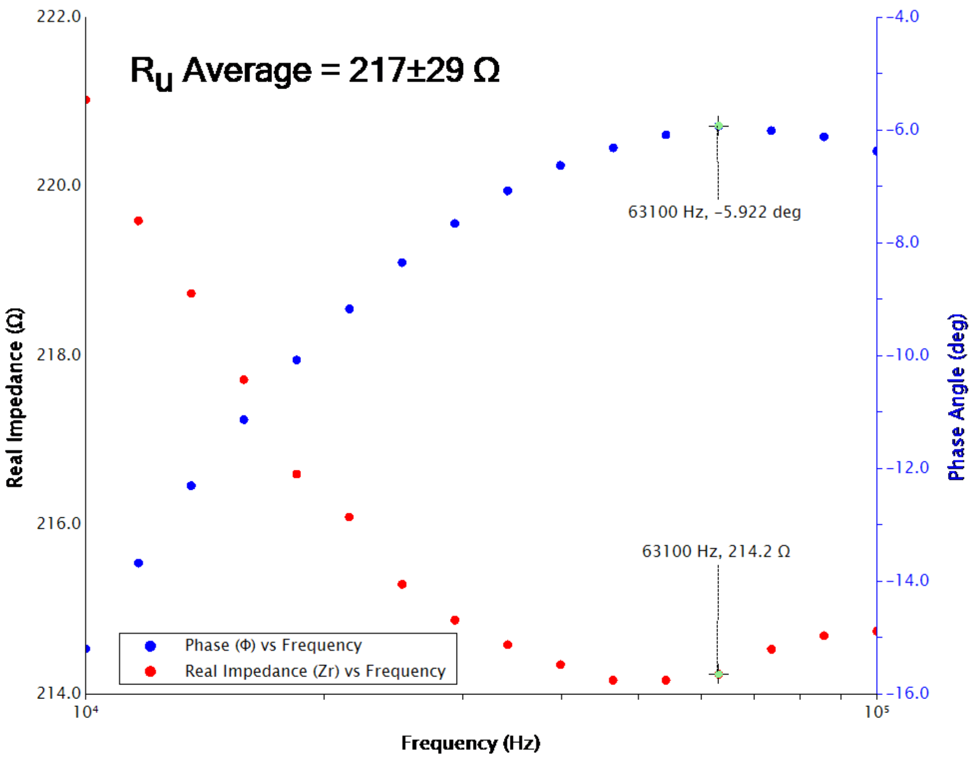

Supplementary Figure 21 | Evaluation of the Uncompensated Resistance. EIS plot of Impendence and phase angle *vs.* frequency for Co NPs electrodeposited on a HOPG substrate allows the determination of uncompensated solution resistance (R_u). At high frequencies the double layer capacitance of the substrate, represented by a capacitor in the Randles cell, behaves as a short, signifying the observed impedance stems from the solution resistance. This model is verified by evaluating the phase angle, which approaches zero when the system consists of only resistive components. Therefore, this pre-programed evaluation included on the Pine instrumentation determines the maximum phase angle and evaluates the solution resistance at that point to give the R_u. Using this evaluation, the uncompensated resistance was determined to be 217 ± 29 Ω over 13 experiments.

Supplementary Table 1 | ICP-MS of various CoNi stoichiometric systems. ICP-MS quantification of bimetallic CoNi NPs fabricated by nanodroplet-mediated electrodeposition in various stoichiometric ratios. Extraction from as-deposited NPs was achieved by dissolution in concentrated nitric acid with subsequent dilution to 5% *v/v*. It is important to note that the total concentration discrepancies between the $Co_{0.5}Ni_{0.5}$ and $Co_{0.25}Ni_{0.75}$/$Co_{0.75}Ni_{0.25}$ samples can be attributed to differences in dilution prior to ICP-MS analysis.

**Alloy**	**Co Concentration ($ng{\cdot}mL^{-1}$)**	**Ni Concentration ($ng{\cdot}mL^{-1}$)**	**Co Mass Fraction**	**Ni Mass Fraction**
$Co_{0.5}Ni_{0.5}$	18019.978	18046.862	0.500	0.500
$Co_{0.25}Ni_{0.75}$	349.709	1083.591	0.244	0.756
$Co_{0.75}Ni_{0.25}$	1006.487	372.168	0.730	0.270

Supplementary Table 2 | Semi-quantitative stoichiometric analysis by EDX. Semi-quantitative elemental composition of HEMG-NP alloy samples from SEM/EDX elemental mapping. Unless otherwise specified, all HEMG-NP samples were prepared with equal mole fraction of metal salt precursor ion components, resulting in NPs with near-equimolar elemental distribution.

	Alloy	Nickel	Cobalt	Vanadium	Chromium	Manganese	Lanthanum	Indium	Gadolinium	Copper
Figure 1c: Stoichiometric Control of Binary CoNi NPs	NiCo	49.0 ± 2.9	51.0 ± 2.9	-	-	-	-	-	-	-
	NiCo (75:25)	72.4 ± 1.0	27.6 ± 1.0	-	-	-	-	-	-	-
	NiCo (25:75)	25.2 ± 1.2	74.8 ± 1.2	-	-	-	-	-	-	-
Figure 2a: Elemental Composition of Low Entropy Metallic Glass NPs with up to Four Components	CrV	-	-	46.4 ± 7.0	53.5 ± 7.0	-	-	-	-	-
	CrCoNi	37.42 ± 0.6	29.8 ± 2.0	-	32.7 ± 1.5	-	-	-	-	-
	CrCoGd	-	26.9 ± 2.2	-	34.9 ± 2.1	-	-	-	38.2 ± 1.0	-
	CrVNiCo	22.8 ± 3.6	28.5 ± 2.9	21.5 ± 2.2	27.2 ± 4.3	-	-	-	-	-
Figure 2b: Elemental Composition of HEMG-NP Systems with up to Eight Principle Components	CrVNiCoMn	19.5 ± 5.1	23.2 ± 3.3	15.5 ± 2.4	25.2 ± 1.4	16.6 ± 1.3	-	-	-	-
	CrVNiCoMnLa	20.4 ±1.6	21.4 ± 1.7	13.5 ± 0.9	17.1 ± 3.0	13.5 ± 1.3	14.0 ± 0.7	-	-	-
	CrNiCoGdCu	18.2 ± 3.5	18.6 ± 2.6	-	20.0 ± 1.7	-	-	-	23.7 ± 5.3	19.6 ± 1.4
	VCrNiCoMnGdCu	11.0 ± 1.5	12.0 ± 1.5	13.0 ± 1.8	15.2 ± 2.2	13.6 ± 3.1	-	-	18.7 ± 2.1	16.4 ± 2.1
	VCrNiCoMnInGdCu	9.7 ± 1.5	11.0 ± 2.4	9.6 ± 1.0	13.2 ± 1.9	13.4 ± 0.9	-	17.0 ± 1.4	14.0 ± 0.7	12.1 ± 1.0

Supplementary Table 3 │ Overpotential, Tafel slope, and mass activity for electrocatalytic materials. Electrocatalytic characterization of CoFeLaNiPt HEMG-NP electrocatalyst, single constituent component electrocatalysts, and HOPG substrate was performed as outlined in the Materials and Methods section. Overpotential values for the OER and the HER were extracted at current density values of j = 10 mA·cm^{-2}. Tafel slope values were extracted from high overpotential regions of the voltammograms for the OER and the HER. Mass activity for individual electrocatalysts were calculated from extracted current values at overpotential values of j = 10 mA·cm^{-2} and normalized to the mass of metal NPs.

	OER			HER		
**Sample**	**Overpotential (V)**	**Tafel Slope (mV•dec^{-1})**	**Mass Activity (mA•µg^{-1})**	**Overpotential (V)**	**Tafel Slope (mV•dec^{-1})**	**Mass Activity (mA•µg^{-1})**
**HOPG**	1.117 ± 0.020	207	-	1.042 ± 0.016	311	-
**Co**	0.383 ± 0.004	90	69.68	0.739 ± 0.008	120	45.54
**Fe**	0.516 ± 0.002	91	66.73	0.688 ± 0.006	164	59.75
**La**	1.049 ± 0.081	208	54.53	1.013 ± 0.036	306	24.41
**Ni**	0.413 ± 0.081	140	122.85	0.810 ± 0.014	131	126.68
**Pt**	0.716 ± 0.009	91	33.55	0.813 ± 0.018	121	5.67
**HEMG**	0.377 ± 0.004	150	-	0.536 ± 0.003	149	-

Supplementary Table 4 | Metal chloride salt compatibility. Metal salt compatibility was screened by mixing 200 µL of stock precursor solutions (100-300 mM in deionized water) and evaluating for colorimetric reactions or precipitate formation. (a) Mixing of H_2PtCl_6 and $SnCl_3$ revealed precipitate formation and thus HEMG-NPs containing both Sn and Pt were not pursued for stoichiometric or electrocatalytic characterization.

	$CoCl_2$	$CrCl_3$	$GdCl_3$	$InCl_3$	$MnCl_2$	$NiCl_2$	H_2PtCl_6	$SnCl_3$	VCl_3	$ZnCl_2$
$CoCl_2$		Compatible	Compatible	Compatible	Compatible	Compatible	Compatible	Compatible	Compatible	Compatible
$CrCl_3$	Compatible		Compatible	Compatible	Compatible	Compatible	Compatible	Compatible	Compatible	Compatible
$GdCl_3$	Compatible	Compatible		Compatible	Compatible	Compatible	Compatible	Compatible	Compatible	Compatible
$InCl_3$	Compatible	Compatible	Compatible		Compatible	Compatible	Compatible	Compatible	Compatible	Compatible
$MnCl_2$	Compatible	Compatible	Compatible	Compatible		Compatible	Compatible	Compatible	Compatible	Compatible
$NiCl_2$	Compatible	Compatible	Compatible	Compatible	Compatible		Compatible	Compatible	Compatible	Compatible
H_2PtCl_6	Compatible	Compatible	Compatible	Compatible	Compatible	Compatible		Incompatible a	Compatible	Compatible
$SnCl_3$	Compatible	Compatible	Compatible	Compatible	Compatible	Compatible	Incompatible a		Compatible	Compatible
VCl_3	Compatible	Compatible	Compatible	Compatible	Compatible	Compatible	Compatible	Compatible		Compatible
$ZnCl_2$	Compatible	Compatible	Compatible	Compatible	Compatible	Compatible	Compatible	Compatible	Compatible	

Compatible Incompatible
